# Increased intracellular persulfide levels attenuate HlyU-mediated hemolysin transcriptional activation in *Vibrio cholerae*

**DOI:** 10.1016/j.jbc.2023.105147

**Published:** 2023-08-09

**Authors:** Cristian M. Pis Diez, Giuliano T. Antelo, Triana N. Dalia, Ankur B. Dalia, David P. Giedroc, Daiana A. Capdevila

**Affiliations:** 1 Fundación Instituto Leloir, Instituto de Investigaciones Bioquímicas de Buenos Aires (IIBBA-CONICET), Buenos Aires, Argentina; 2 Department of Chemistry, Indiana University, Bloomington, Indiana, USA; 3 Department of Biology, Indiana University, Bloomington, Indiana, USA

**Keywords:** bacterial pathogenesis, bacterial toxin, bacterial transcription, host–pathogen interaction, homocysteine, sulfur, thiol, transcription regulation, *Vibrio cholerae*

## Abstract

The vertebrate host's immune system and resident commensal bacteria deploy a range of highly reactive small molecules that provide a barrier against infections by microbial pathogens. Gut pathogens, such as *Vibrio cholerae*, sense and respond to these stressors by modulating the expression of exotoxins that are crucial for colonization. Here, we employ mass spectrometry–based profiling, metabolomics, expression assays, and biophysical approaches to show that transcriptional activation of the hemolysin gene *hlyA* in *V. cholerae* is regulated by intracellular forms of sulfur with sulfur–sulfur bonds, termed reactive sulfur species (RSS). We first present a comprehensive sequence similarity network analysis of the arsenic repressor superfamily of transcriptional regulators, where RSS and hydrogen peroxide sensors segregate into distinct clusters of sequences. We show that HlyU, transcriptional activator of *hlyA* in *V. cholerae*, belongs to the RSS-sensing cluster and readily reacts with organic persulfides, showing no reactivity or DNA dissociation following treatment with glutathione disulfide or hydrogen peroxide. Surprisingly, in *V. cholerae* cell cultures, both sulfide and peroxide treatment downregulate HlyU-dependent transcriptional activation of *hlyA*. However, RSS metabolite profiling shows that both sulfide and peroxide treatment raise the endogenous inorganic sulfide and disulfide levels to a similar extent, accounting for this crosstalk, and confirming that *V. cholerae* attenuates HlyU-mediated activation of *hlyA* in a specific response to intracellular RSS. These findings provide new evidence that gut pathogens may harness RSS-sensing as an evolutionary adaptation that allows them to overcome the gut inflammatory response by modulating the expression of exotoxins.

Many bacterial pathogens secrete diverse protein toxins to disrupt host defense systems, which are expressed with precise spatiotemporal regulation, since untimely toxin secretion can be detrimental to the invading pathogens (1, 2). Such is the case with *Vibrio cholerae*, the major causative agent of the severe diarrheal disease, cholera. In this organism, the expression of various enteric exotoxins is under exquisite control of distinct transcriptional regulators that trigger their expression upon attachment to the small intestine epithelium surface, enabling efficient colonization (3). Cholera toxin (CT), the major virulence factor responsible for cholera pathogenesis, and other accessory toxins and virulence factors (*e.g.*, Ace, Zot, TCP) are primarily regulated by the transcriptional activator ToxT (4). Beyond ToxT-activated genes, pathogenic strains of *V. cholerae* produce several additional accessory toxins (2) such as the extracellular pore-forming toxin hemolysin (HlyA) (5), which is implicated in pathogenesis, particularly, in those strains that lack CT (6). *hlyA* is activated by HlyU and repressed by the quorum-sensing regulator HapR and the iron uptake repressor Fur (7). While HapR and Fur link quorum sensing (8) and the cellular iron status (9) to virulence gene regulation, which is likely advantageous in the human host, the signals that modulate HlyU-dependent activation of HlyA in *V. cholerae* and other exotoxins in other *Vibrio species* remain unresolved (10, 11, 12, 13, 14). Here, we present a biochemical and functional characterization of HlyU-mediated responses toward microenvironmental signals thought to be present in the gut that may impact hemolysin expression of *V*. *cholerae* and other exotoxins in pathogenic *Vibrio* species (11, 12, 13, 15).

To cause cholera, *V. cholerae* must effectively colonize the small intestine, overcoming many host-derived stressors (3), such as the low pH of the human stomach, liver-derived bile, and antimicrobial peptides in the intestinal lumen. More recent work shows that gut pathogens must also adapt to hydrogen sulfide stress imposed by the host or gut microbiota (16), which involves an increase in bile acid (taurocholic acid)–derived hydrogen sulfide (H_2_S) and potentially more oxidized sulfur–bonded sulfur compounds (namely “sulfane” sulfur or reactive sulfur species [RSS]) (17, 18, 19). Although gut pathogen adaptation to such stressors remains understudied, the protein machinery charged with H_2_S/RSS remediation and, ultimately, efflux has been described for many bacterial pathogens and free-living bacteria (20, 21, 22, 23, 24, 25). There is now considerable evidence that beneficial levels of H_2_S and low-molecular weight thiol persulfides can protect bacteria from oxidative stress that arises from inflammatory responses (26, 27, 28), as well as antibiotics (29, 30, 31). Recently, it has been shown that *V*. cholerae produces endogenous H_2_S, which decreases its susceptibility to hydrogen peroxide (H_2_O_2_) in both *in vitro* and *in vivo* adult mice models (32). Thus, beyond exposure to exogenous H_2_S (16), intracellular H_2_S/RSS levels in *V. cholerae* also depend on the synthesis of endogenous H_2_S from cysteine metabolism. We speculate that exogenous and endogenous levels of H_2_S may serve as additional microenvironmental cues that impact small intestine colonization dynamics and toxin gene expression.

H_2_S is an important signaling molecule for gut bacteria. However, little is known about the intracellular concentration of the components of the RSS pool, something particularly true for *V. cholerae*. This encompasses organic and inorganic molecules containing sulfur in an oxidation state higher than H_2_S while also containing sulfur atoms covalently bonded to other sulfur atoms, often in polysulfur chains and collectively termed “sulfane” sulfur (19). These species are both responsible for the beneficial biological properties of H_2_S, as well as its toxicity at least in part, as RSS can effectively modify catalytic residues in proteins, often negatively impacting their activity (22, 33, 34, 35). Signaling by RSS is achieved predominantly *via* a posttranslational modification of cysteine residues in proteins, often referred to as *S*-sulfuration, persulfidation, or *S*-sulfhydration (34). Thus, the speciation of “sulfane” sulfur inside the cells—meaning the cellular concentrations of all organic, inorganic, and protein species—must be controlled. Bacteria maintain H_2_S/RSS homeostasis by expressing persulfide-sensing transcriptional regulators, whose regulons generally encode for a subset of common downstream H_2_S detoxification genes (25, 36, 37). Although the mechanisms that define H_2_S/RSS homeostasis in bacteria have been described for several human pathogens (18, 22, 33), little is known about how *V*. *cholerae* responds to the increasing H_2_S/RSS and how these reactive species affect pathogen metabolism and, ultimately, gut colonization.

The described persulfide-sensing transcriptional regulators in bacteria belong to three structurally unrelated protein families, namely the arsenic repressor (ArsR), copper-sensitive operon repressor (CsoR), and Fis superfamilies. They all harness dithiol chemistry to form either disulfide or polysulfide bridges between reactive cysteine (Cys) residues that allow for the transcription of sulfide metabolism genes, either by transcriptional derepression or by RNA polymerase recruitment and transcriptional activation (25, 33, 36, 37, 38). Beyond *bona fide* persulfide-sensing transcriptional regulators that control the expression of at least one sulfide metabolism gene, other dithiol-harboring transcriptional regulators have been reported to react and elicit transcriptional responses in the presence of persulfides and/or polysulfides (39, 40, 41, 42). To what extent these sensors are truly specific to persulfides and can be distinguished from other redox sensors that readily react with H_2_O_2_ *in vitro* remains a matter of debate despite the detailed structural information available (36, 37). Nevertheless, it is interesting to evaluate persulfide sensing as an evolutionary advantage for human pathogens, as these species are prevalent and biosynthesized in certain tissues by the host or host-resident microbiota, notably in the gut (43).

To date, no persulfide-sensing transcriptional regulator has been identified in *Vibrio* strains. Thus we aimed to predict putative regulators that would respond to these species by sequence homology to ArsR, CsoR, and Fis family proteins encoded in the *V. cholerae* genome. While *V. cholerae* strains do not generally encode for CsoR (37) or FisR family proteins (44), they encode at least two ArsR family proteins, one of which is HlyU and the other one annotated as BigR (biofilm repressor) in some strains (45). Both proteins conserve the Cys pair that functions as the sensing site in *Rhodobacter capsulatus* SqrR and *Acinetobacter baumanii* BigR, two well-characterized persulfide-sensing transcriptional regulators from the ArsR family ((20, 33, 36)). These RSS-sensing repressors react with both organic and inorganic persulfides, but not with disulfides or peroxides, leading to the formation of a polysulfide bridge between the cysteines (36), and are readily distinguished from other ArsR family members that harbor H_2_O_2_-sensing proximal cysteine site (46). While *Vc*BigR and other ArsR family proteins in *V. cholerae* remain uncharacterized, the structure of *Vc*HlyU is known and the role of cysteine oxidation in inhibiting DNA operator binding has been previously reported (47). However, it remains unclear whether HlyU senses H_2_O_2_ levels in its local environment since Cys sulfenylation may not be prevalent in the gastrointestinal tract due to the microaerobic or anaerobic conditions. In this context, the sequence similarities between HlyU and previously characterized *bona fide* persulfide sensors strongly motivate experiments capable of defining the chemical specificity of this transcriptional regulator and, ultimately, understand the molecular basis of how HlyU oxidative modifications prevent the initiation of the virulence cascade in *Vibrio* spp.

In this study, we first use a comprehensive sequence similarity network (SSN) analysis of >150,000 sequences to identify distinct clusters of RSS-sensitive and H_2_O_2_-sensitive regulators, which reveals that HlyU is most closely related to previously characterized persulfide sensors (36). Next, we investigated the reactivity of HlyU using *in vitro* mass spectrometry (MS) and fluorescence anisotropy, which show that HlyU reacts with organic persulfides to form a tetrasulfide bridge between its two cysteines, abrogating DNA binding. In striking contrast, disulfides and peroxides do not react with HlyU nor do they impact DNA binding. We found that the exogenous treatment of *V. cholerae* cells with either sulfide (Na_2_S) or H_2_O_2_ attenuates HlyU-dependent activation of *hlyA*, downregulating *hlyA* transcription likely through transcriptional silencing by the nucleoprotein H-NS (48). Quantitative RSS metabolite profiling experiments reveal that both treatments result in an increase in the levels of inorganic persulfides, thus reconciling the *in vitro* and *in vivo* results. Together, these findings suggest that persulfides function as the cognate regulator of HlyU-regulated gene expression, thus uncovering a new role for RSS sensing in exotoxin expression in a major enteric pathogen.

## Results

### SSN analysis of the ArsR superfamily suggests that HlyU is an RSS-sensing transcriptional regulator

**Figure 1:**
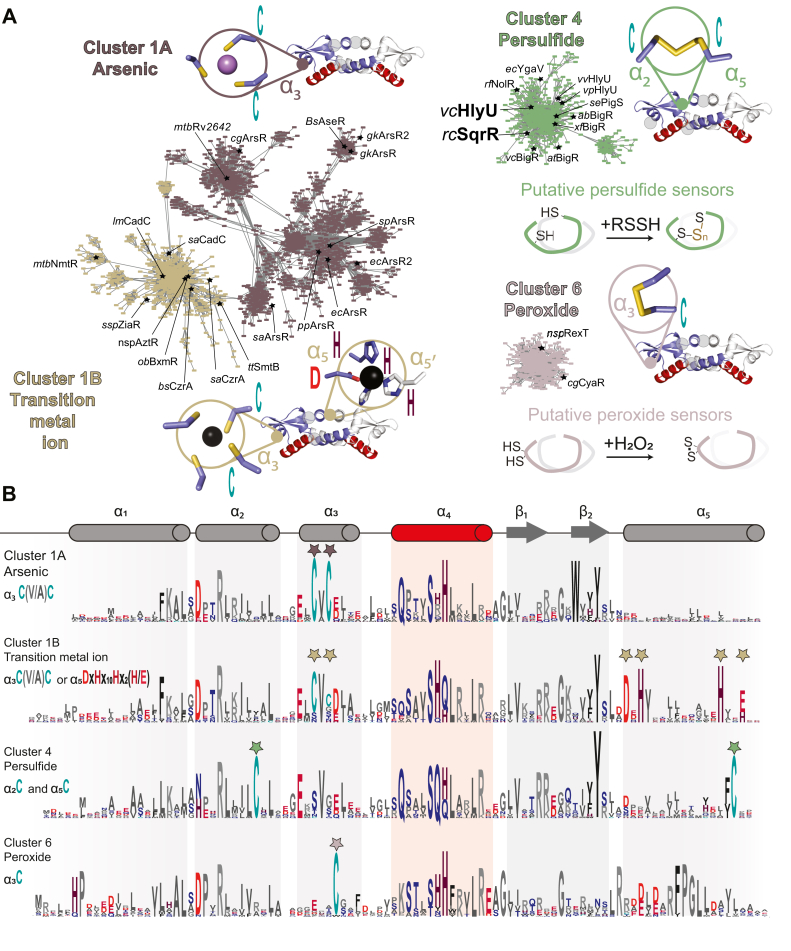
**Sequence similarity network analysis of the ArsR superfamily of bacterial repressors.***A*, results of an SSN clustering analysis of 168,163 unique sequences belonging to the Pfam PF01022 and Interpro IPR001845 using genomic enzymology tools and visualized using Cytoscape. Clusters were functionally annotated as arsenic, transition metal ions, persulfide, or hydrogen peroxide sensors are presented here, along with a representation of the inducer recognition site as an inset mapped onto a representative ArsR structure (PDB codes: 6j0e, 2kjc, 6o8n, 7txm, and 6o8l, respectively). All the determined main clusters are presented in Fig. S1. All clusters are designated by a number and ranked according to the number of unique sequences (Table S2), color-coded. Each node corresponds to sequences that are 50% identical over 80% of the sequence, using an alignment score of 22 (see Experimental procedures). Functionally characterized members in each are indicated with species name and trivial name, except for *vc*BigR, which is included for clarity and has not been characterized biochemically. *B*, sequence logo representations of sequence conservation defined by the indicated cluster of sequences derived from *panel A*. The residues that coordinate metals/metalloid ions or undergo redox chemistry in ArsR are marked by *stars*. We note that coordinating residues in variable regions in the N terminus or C terminus do not always appear in the sequence logos. ArsR, arsenic repressor; SSN, sequence similarity network.

ArsR superfamily proteins are compact homodimeric DNA-binding proteins characterized by a core α1-α2-α3-α4-β1-β2-α5 secondary structure; some members contain extensions on either or both N- and C-terminal sides of this motif, and if α-helical are denoted the α0 and α6 helices, respectively, to facilitate sequence comparisons (49, 50). The helix-turn-helix motif that mediates DNA binding is the α3-α4 segment that engages successive DNA major grooves, with a degenerate tetrapeptide sequence in α4 (Fig. S1*A*) that enforces specificity for a particular DNA operator sequence (51). The β1-β2 wing extends from the periphery of the dimer and may mediate interactions with the immediately adjacent minor grooves. Early work on ArsR family proteins suggested that the >3000 distinct members of this family were mostly metal ion or metalloid-specific regulators (52, 53, 54). However, it has become increasingly clear that this is not the case (50, 55) as many recently described regulators have been shown to respond to redox-active small molecules (20, 24, 46) or may lack inducer binding sites altogether (56, 57, 58). In order to evaluate these sequence relationships and the extent to which these differences in inducer recognition sites are captured in a large superfamily with low pairwise sequence conservation, we performed an SSN analysis (59) of 168,163 unique entries from the Pfam PF01022 and Interpro IPR001845 datasets (13,879 sequences that are <50% identical over 80% of the sequence; UNIREF50 clusters). Functionally characterized members in each cluster suggest that individual clusters may represent groups of proteins that share the same chemical inducer (Figs. 1*A* and S1*A* and Table S2), which is further supported by the conservation of inducer recognition residues in well-described distinct structural motifs within each SSN cluster (Fig. 1*B*) (50).

The largest SSN cluster that appears at this level of sequence segregation (see Experimental procedures) is SSN cluster 1, which can be divided into two large subclusters, denoted here as 1A and 1B. These sequences share a highly conserved CXC motif in the third helix (α3) (Fig. 1*A*, *dark* brown) and are distinguished from one another by the absence (subcluster 1A) or presence (subcluster 1B) of an additional inducer recognition site known to bind transition metals (Fig. 1*A*; gold) (50). Cluster 1, in fact, contains the vast majority of described metalloregulators to date, that sense either biological transition metals, for example, Cu(I), Zn(II), Ni(II), or Co(II) and heavy metal xenobiotics Cd(II) and Pb(II) (subcluster 1B) or trivalent As(III)/Sb(III) (subcluster 1A). The exceptions are the sequences compiled in SSN cluster 21, representative of the Ni(II) sensor SrnR (58) and those in SSN cluster 22, representative of the Cd(II) sensor CmtR, with metal coordinating ligands derived from the DNA-binding helix α4 (Fig. S1*B*) (60, 61). Subcluster 1B contains all historically characterized, canonical As(III) sensors harboring a C-(V/A)-C motif that coordinates As(III), while cluster 5 includes many more recently described atypical As(III) sensors that feature trigonal Cys coordination with all metal ligands derived from α5 (Fig. S1*B*) (62).

The remainder of the SSN clusters represent proteins that are not obviously metal ion or metalloid-sensing regulators. SSN clusters 2 and 3 (Fig. S1*B*) remain largely uncharacterized and contain members involved in some way in the hypoxic response in *Mycobacterium tuberculosis* (Rv2034 and Rv0081, respectively, Table S2) (57, 63). SSN cluster 4 encompasses all known RSS or persulfide sensors characterized by a pair of cysteines in the α2 and α5 helices, which form an intraprotomer polysulfide bridge when presented with “sulfane” sulfur donors (33, 36). As expected from a previous SSN (46), cluster 4 sequences are readily distinguished from those in SSN cluster 6, which contains a recently characterized dithiol protein, RexT, that reacts with H_2_O_2_ *via* two proximal Cys residues in α3. Overall, our SSN analysis suggests that ArsR proteins that lack a metal-binding site can be readily distinguished and may indeed harness certain degree of chemical specificity of distinct dithiol sites. Moreover, the two ArsR protein encoded by *V. cholerae*, HlyU (locus tag VC_0678), and BigR (VC_0642) are members of SSN cluster 4, thus suggesting they may respond primarily to inorganic and organic persulfides, and not to hypoxia as described for proteins in SSN clusters 2 and 3, nor H_2_O_2_ as it has been described for SSN cluster 6.

These functional assignments are further supported by a genome neighborhood analysis, based on the premise that in bacteria, regulatory and functional genes dedicated to a particular task tend to form gene clusters in the chromosome. While the genes encoding the metalloregulators in SSN clusters 1A, 21, and 22 are generally nearby one or more genes encoding a metal ion transporter, the neighboring genes of As(III)-dependent repressors (clusters 1B and 5) encode for arsenate-transferring proteins and organoarsenic transporters (Fig. S2). Similarly, known persulfide-sensing cluster 4 regulators genomically colocalize with genes encoding sulfurtransferases or rhodaneses and inorganic sulfur transporters (21, 22, 33). In contrast, genes encoding peroxide-sensing SSN cluster 6 regulators are generally nearby genes encoding NADH oxidoreductases. A more comprehensive analysis of the regulons of biochemically characterized SSN cluster 4 persulfide–sensing repressors suggests that exotoxin expression in *Vibrio* ssp., and biofilm production and antibiotic biosynthesis in others is linked in some way to RSS-sensing in cells, a remarkably diverse collection of adaptive responses that are likely tuned to bacterial lifestyle and environmental needs. In this context, we characterize *V. cholerae* HlyU, the positive regulator of hemolysin production. The inducer selectivity of HlyU remains undefined, although previous work implicates reactive oxygen species in this role, in striking contrast to the implications of SSN analysis which places HlyU in SSN cluster 4 (14).

### HlyU reacts exclusively with persulfides to form a tetrasulfide bridge that leads to reversible DNA dissociation

**Figure 2:**
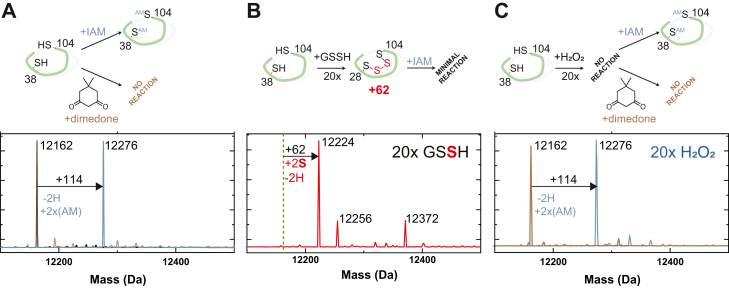
**LC-ESI-MS analysis of HlyU *in vitro* reactivity upon the addition of GSSH or H**_**2**_**O**_**2.**_*A*, HlyU reacts with IAM but not with dimedone, showing that the protein is fully reduced. *B*, HlyU reacts with GSSH to form a tetrasulfide link between its two cysteines, as seen in the mass shift of +62, corresponding to the addition of two sulfur atoms and the subtraction of two hydrogens. *C*, HlyU does not react with H_2_O_2_, as seen by the absence of a peak corresponding to a dimedone adduct (127). IAM, iodoacetamide.

To evaluate biochemically if sulfide signaling through persulfides impacts exotoxin expression in *V. cholerae* through a HlyU-dependent mechanism, we determined the ability of HlyU to distinguish between persulfides and other nonsulfur containing oxidants. We exploited an MS-based, anaerobic assay (36, 64) to determine the reactivity of HlyU toward redox-active small molecules in a time-resolved manner. In this assay, we employ quantitative capping by excess iodoacetamide (IAM) in the absence of denaturing agents, as confirmation that the protein is fully reduced. Excess IAM is removed in less than 5 min after quenching to prevent undesired chemistry of the capping agent (65) (Fig. 2*A*). Consistent with expectations from the placement of HlyU among other persulfide sensors in SSN cluster 4 (36), we observed no change in the mass spectrum upon IAM capping, following a 1-h incubation with a 20-fold molar excess of H_2_O_2_ and glutathione disulfide (Figs. 2, *A* and *C* and S3). In contrast, reduced HlyU readily reacts with inorganic (Na_2_S_4_) and organic (glutathione persulfide, GSSH; cysteine persulfide, CSSH; homocysteine persulfide, hCSSH) persulfides, shifting the mass distribution to a +62-Da species, consistent with an intramolecular (intraprotomer) tetrasulfide crosslink between the conserved Cys38 and Cys101 residues (Figs. 2, *A* and *B* and S3). In addition to the tetrasulfide linkage, a significant amount of pentasulfide is formed. Interestingly, HlyU polysulfides differ from previously reported experiments carried out with SqrR and *Ab*BigR, in that they can be partially capped with IAM. This suggests that the polysulfide links between the two cysteines are in equilibrium with “open” hydropersulfide or hydropolysulfide forms. Moreover, to rule out the formation of the sulfenic acid on Cys38 previously captured by crystallography in air in absence of a reducing agent (47), we performed overnight aerobic reactions with IAM or 5,5-dimethylcyclohexane-1,3-dione (dimedone). These experiments confirm that both Cys remain reduced when treated with H_2_O_2_ and are capable only of forming a tetrasulfide bridge when treated with “sulfane” sulfur donors (Fig. 2, *B* and *C*).

**Figure 3:**
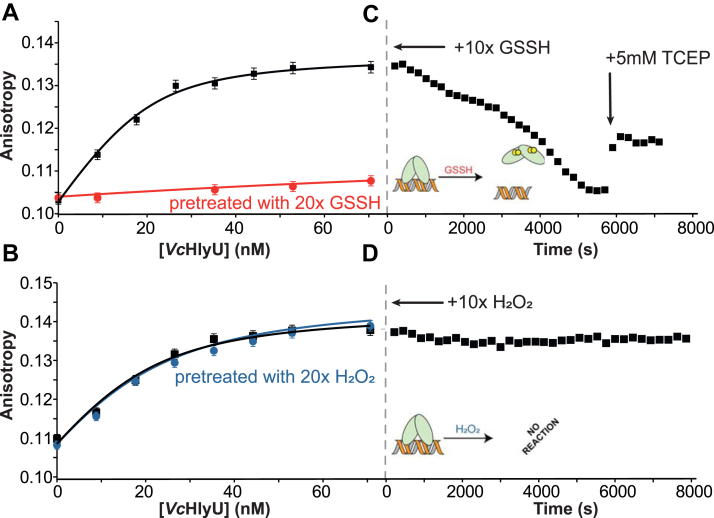
**DNA-binding isotherms of *Vc*HlyU over its DNA operator in different oxidation states at 100 mM NaCl.***A*, reduced (*black*) *versus* GSSH pretreated (*red*) and (*B*) reduced (*black*) *versus* H_2_O_2_ pretreated (*blue*). Anisotropy changes of the fluorescein-labeled HlyO operator with *Vc*HlyU after addition of a 10-fold excess of either (*C*) GSSH or (*D*) H_2_O_2_. After addition of oxidant the anisotropy was followed over time until a new equilibrium condition was reached. Then a final concentration of 5 mM TCEP was added to the solution to test the reversibility of the oxidation. DNA-binding isotherms were obtained using the DynaFit software after a global fit of at least two replicates in each case. TCEP, tris(2-carboxyethyl)phosphine.

We further confirmed the specificity of persulfide-induced regulation by measuring the DNA-binding affinities of *Vc*HlyU for a known DNA operator in the hemolysin promoter (7). These quantitative fluorescence anisotropy-based DNA-binding experiments reveal that reduced HlyU binds to the operator upstream of HlyA with a 0.40 × 10^9^ M^−1^ affinity constant, which is comparable to other ArsR proteins. This affinity remains unchanged in the absence of reducing agent (Fig. 3*A*, Table 1). H_2_O_2_ pretreatment also leads to no change in DNA-binding affinity, while GSSH pretreatment yields a protein with virtually no DNA-binding activity (Fig. 3, *A* and *B*). Moreover, upon titrating HlyU to saturation in the absence of reducing agent, it is possible to fully dissociate the protein from the DNA with the addition of a 10-fold excess of “sulfane” sulfur over HlyU in a GSSH-containing mixture, as indicated by the decrease in anisotropy to that of the free DNA value. This dissociation is quite slow, however, which might suggest that formation of the HlyU tetrasulfide may be protein catalyzed in cells (66). DNA binding is only partially restored upon addition of a reducing agent (Fig. 3*C*); the reasons for this are unknown, but might be indicative of overoxidation or that full reduction requires a protein depersulfidase (67). In striking contrast, addition of 10-fold of H_2_O_2_ to DNA-bound HlyU leads to no change in the anisotropy (Fig. 3*D*), providing further evidence that increased levels of H_2_O_2_ do not directly impact DNA binding.

**Table 1:** DNA-binding affinities obtained for various forms of HlyU

HlyU pretreatment	*K*_a_ [× 10^9^ M^−1^]^a^
Reduced	0.40 ± 0.08 (*n* = 4)
GSSH treated	0.009 ± 0.005 (*n* = 2)
H_2_O_2_ treated	0.19 ± 0.03 (*n* = 3)

a Experimental conditions: 25 mM Hepes, pH = 7, 100 mM NaCl, 1 mM EDTA, 25 °C. K_a_ and errors were obtained from the global fit of all replicates using DynaFit software. The treatment corresponds to a 5-fold excess relative to the protein subunit concentration.

Our MS-based reactivity assays and fluorescence anisotropy–based DNA-binding experiments confirm that a posttranslational thiol modification on HlyU negatively impacts DNA binding and that these modifications are selective toward “sulfane” sulfur compounds. To gain further insights on the structural impact of persulfide and peroxide treatment of HlyU homodimer, we used solution NMR as a probe for conformational changes that lead to DNA dissociation. ^1^H-^15^N heteronuclear single quantum coherence spectra were acquired for HlyU dimer in the reduced, H_2_O_2_- and GSSH-treated states (Fig. S4). While the spectra obtained for HlyU in the reduced and H_2_O_2_ treated are essentially identical, GSSH treatment introduces significant perturbations in the spectrum, consistent with a well-folded dimer that is characterized by a distinct structure or distinct dynamics, which ultimately leads to DNA dissociation. To further explore the conformational changes induced by the tetrasulfide bond formation, we performed a series of CD experiments at different temperatures to determine the stability of the secondary structure in the reduced, disulfide (diamide-treated (36)), and tetrasulfide crosslinked forms. The CD spectrum from HlyU is typical of a protein with significant secondary structure and a prevalence of α-helices, with a positive band <200 nm and negative signals at 210 and 218 nm (Fig. S5*A*). Reduced HlyU has a melting temperature (*T*_m_) of 65 °C (68). Formation of the tetrasulfide decreases the native conformation stability by a small extent (*T*_m_ = 60 °C), while disulfide bond formation yields a far less stable form (*T*_m_ =52 °C) (Fig. S5*B*). The comparatively lower stability of HlyU disulfide form is consistent with the observation of a high-structural frustration in the disulfide-bonded structure of SqrR (36) and provides insights into the low reactivity of HlyU with oxidants that would ultimately lead only to a disulfide-bonded form.

### Exogenous H_2_O_2_ and sulfide treatment of *V. cholerae* cell cultures impair HlyU-mediated p*hlyA* activation

**Figure 4:**
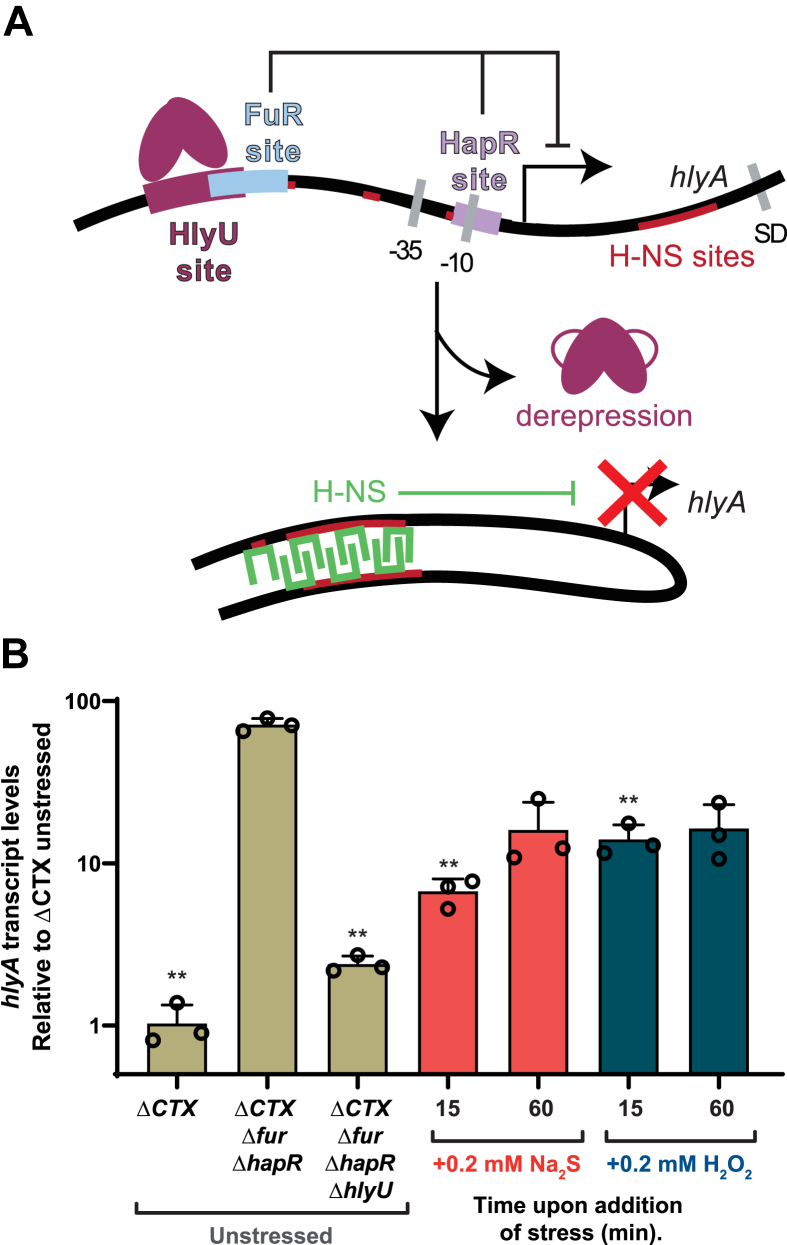
**HlyU mediated p*****hlyA*** **activation followed by quantitative RT-PCR.***A*, model of the mechanism of HlyU regulation of the *hlyA* gene, HlyU DNA dissociation leads to H-NS (*green*)–mediated repression. *B*, quantitative RT-PCR performed over a Δ*CTX* Δ*fur* Δ*hapR Vibrio cholerae* strain with the addition of Na_2_S or H_2_O_2_. The *bar chart* shows the fold changes of induction of *Vc hlyA* after addition of Na_2_S (*red*) and H_2_O_2_ (*blue*), with transcript values normalized relative to the transcription level of *recA*. The values correspond to transcript levels relative to unstressed (UN) (*middle sand bar*) and are shown as mean ± SD from three replicate cultures. Statistical significance was established using a paired *t* test relative to UN under the same conditions (∗∗*p* < 0.01, ∗*p* < 0.05). *Lines* on the top of the chart show statistical significance relative to Δ*CTX* Δ*fur* Δ*hapR V. cholerae* mutant strain UN.

Given that HlyU has properties of *bona fide* RSS–sensing repressor, we next aimed to determine the impact of cellular persulfides on HlyU-dependent regulation of hemolysin expression. Hemolysin expression is complex and known to be regulated by HapR, Fur, and HlyU in *V. cholerae* El Tor Serogroup O1 (7) (Fig. 4*A*). HlyU is somewhat unique among the ArsR family members in that instead of functioning as a repressor that inhibits the binding of RNA polymerase, it binds to the *hlyA* promoter and activates its transcription (50). The HlyU-dependent activation mechanism is best described in *Vibrio vulnificus* (69). Here, HlyU employs a “counter-silencing” mechanism and displaces the global transcriptional repressor in Gram-negative bacteria, H-NS, from the two most upstream sites in the *hlyA* promoter, relieving transcriptional repression and leading to its activation upon HlyU recruitment. In *V. cholerae*, the location of HapR, Fur, and HlyU binding has been described (50), and it has been shown that H-NS occupancy at the *hlyA* promoter is diminished by HlyU overexpression (70). However, the precise sites of H-NS binding are not known and the existing chromatin immunoprecipitation assays with sequencing data suggest that H-NS may also bind *hlyA*-coding regions (71). Thus, we first developed an assay where we could distinguish HlyU-mediated activation, while decoupling hemolysin gene expression from quorum sensing and other environmental queues that impact these other transcriptional regulators involved in hemolysin regulation.

We first used a P_hlya_-*GFP* transcriptional reporter in different strain backgrounds to interrogate HlyU activity (Fig. S6). Consistent with previous findings (50), *ΔhlyU* alone did not decrease GFP fluorescence compared to parent as HlyA expression is strongly repressed by Fur and HapR (Fig. 4*A*). As a result of this repression, HlyU-mediated *hlyA* expression is the highest in a *Δfur ΔhapR* background (Fig. S6*A*). This is also the case when *hlyA* transcript levels are followed by quantitative real-time PCR (qRT-PCR), and this activation is dependent on HlyU (Fig. 4*B*, sand-shaded bars). Furthermore, an H-NS deletion in the *Δfur ΔhapR* background provides additional support for the idea that the HlyU-dependent mechanism for P_h__l__y__A_ activation is eviction of H-NS (70) (Fig. S6*B*). However, it should be noted that the *Δhns* strains exhibit a very strong biofilm phenotype, which complicates the fluorescence measurement (72). Interestingly, the difference in transcript levels of the *hlyA* and *gfp* genes that share the same P_h__l__y__A_ promoter suggest that HlyU-mediated activation may be enhanced by immediately adjacent regions, either downstream or upstream of the promoter, as the magnitude of transcriptional activation of *hlyA* is at least 20-fold higher in its native context relative to that of the *gfp* reporter, which only increases by 50% when HlyU is expressed (Figs. 4*B* and S7). This is not unexpected given the H-NS protection of P_h__l__y__A_ includes not only the promoter region but also at least 700 bp that encode for the HlyA protein (71).

We therefore elected to measure native *hlyA* transcript levels in a *Δfur ΔhapR* background, as it best recapitulates the H-NS eviction mechanism while isolating this event from Fur- and HapR-dependent effects. We next monitored HlyU-mediated transcriptional activation, following acute Na_2_S and H_2_O_2_ stress (0.2 mM) in LB media at *A*_600_≈0.2 after 15 and 60 min, as analogous persulfide sensors in other organisms are characterized by an acute phase transcriptional response (37). We used qRT-PCR to assess induction of *hlyA*, *gfp* (of the P_hlya_-GFP reporter), and *hlyU* mRNA expression. We observe a robust sulfide- and peroxide-inducible inhibition of the activation of *hlyA* expression (Fig. 4*B*, red and blue bars, respectively), while *gfp* and *hlyU* do not show significant differences upon stressor addition (Fig. S7). Exogenous treatment with these species is expected to trigger oxidative and sulfide stress signaling inside cells as they are both in equilibrium with membrane permeable species, H_2_S and neutral H_2_O_2_ respectively. Thus, our results suggest that an acute increase in intracellular levels of either H_2_S or H_2_O_2_ can downregulate *hlyA* transcription in a HlyU-mediated mechanism that likely involves H-NS polymerization on both coding and noncoding regions of the *hlyA* region. This crosstalk contrasts with the high degree of inducer specificity of HlyU suggested by the *in vitro* chemical reactivity, DNA-binding and NMR experiments. Nonetheless, our results show for the first time that HlyU-dependent activation of the *hlyA* operon can be modulated in response to exogenous stressors, including H_2_S.

### RSS metabolite profiling experiments

A change in the cellular levels of persulfidated low-molecular weight thiols (LMWTs) upon exogenous hydrogen sulfide stress is a robust biomarker for intracellular sulfide accumulation, having ultimately led to the identification of distinct features of thiols/RSS homeostasis in *Staphylococcus aureus* (73), *Enterococcus faecalis* (22), *Acinetobacter baumannii* (33), *Salmonella* (74) and, more recently, *Streptococcus pneumoniae* (75) and *R. capsulatus* (76). Although the mechanistic details remain lacking, an emerging picture is that H_2_S is converted to thiol persulfides either *via* the enzymatic activity of sulfide:quinone oxidoreductase (77), or through other enzymatic (66) or nonenzymatic processes (78); this, in turn, leads to a transient increase in persulfidation of small molecule and proteome-derived thiols (66). The degree and identity of small molecule persulfidation, that is, speciation of the LMWT pool, depends to some extent on relative abundance of these species inside the cells (79). Changes in LMWT persulfidation have also been observed by endogenous production of H_2_S as deletion of the enzymes that biosynthesize it decreases the levels of persulfidation of LMWT, although the effect is small (33). The endogenous production of H_2_S can be also triggered by exogenous treatment with reactive oxygen species, such as H_2_O_2_. This H_2_O_2_-enhanced H_2_S endogenous production has been reported in *V. cholerae* and it has been shown that is has a critical role in cytoprotection against oxidative stress (32). However, the role of this H_2_S in signaling in *V. cholerae* is largely unknown, as is any quantitative information on LMWTs and LMW RSS speciation in cells.

**Figure 5:**
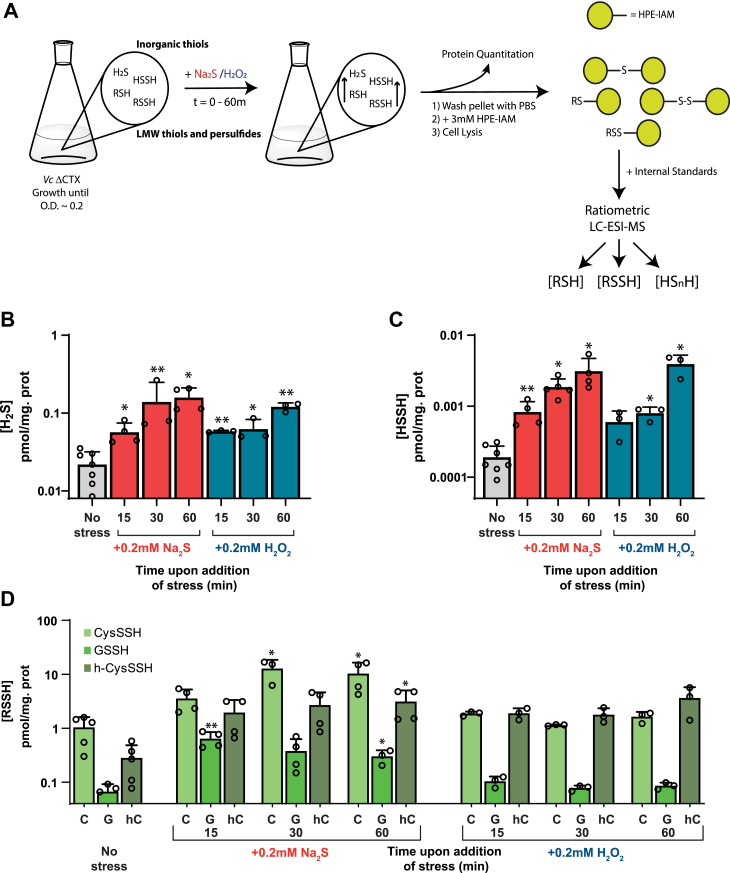
**LMWT and LMW persulfide metabolite profiling of *Vibrio cholerae* strains.** *A*, cartoon representation of the scheme for LMWT and LMW persulfide profiling. Growth of a ΔCTX *Vibrio cholerae* strain until *A* of ∼0.2 is followed by the addition of Na_2_S or H_2_O_2_ to a final concentration of 0.2 mM. Cultures were centrifuged at 0 (prior to addition of the stressor), 15, 30, and 60 min. In all cases 1 ml of sample was withdrawn for protein quantitation. The metabolite profiling was generally carried out using HPE-IAM as labeling agent. The ratiometric LC-ESI-MS experiments were performed with the dilution of isotopically labeled internal standards of known concentration, which were used for quantitation of the organic and inorganic species. *B*, endogenous concentrations of hydrogen sulfide before and after addition of stress (Na_2_S, *red*; H_2_O_2_, *blue*) to midlog-phase cultures. *C*, endogenous concentrations of hydrogen disulfide before and after addition of stress (Na_2_S, *red*; H_2_O_2_, *blue*) to midlog-phase cultures. *D*, endogenous concentrations of CysSSH, GSSH, and h-CysSSH before and after addition of stress at different timepoints to midlog-phase cultures. Statistical significance was established using a paired *t* test relative to UN under the same conditions (∗∗*p* < 0.01, ∗*p* < 0.05). HPE, β-hydroxyphenyl-ethyl; IAM, iodoacetamide.

Given the paradoxical findings that HlyU does not react with H_2_O_2_ *in vitro*, yet H_2_O_2_ results in attenuation of HlyU-dependent activation, we hypothesized that both treatments trigger a change in reactive sulfur speciation that leads to HlyU modification. To test this hypothesis, we first employed an isotope dilution, electrophile trapping method to estimate the endogenous levels of the major cellular thiols, thiol persulfides, and inorganic sulfide and “sulfane” sulfur–bonded species in cell lysates from mid-exponential phase cells (33) (Fig. 5*A*). Consistent with previous reports for *Vibrio* strains (80, 81), we find that cysteine and GSH are the major cellular thiols, followed by homocysteine at a concentration ≈5- to 10-fold lower (Fig. S8*A*). Basal H_2_S levels are much lower than that of the other thiols (Fig. 5*B*). Some β-(4-hydroxyphenyl)ethyl (HPE)-IAM capped thiols and persulfides (*e.g.*, coA) are not easily quantified in the same chromatographic run and their concentrations were determined using monobromobimane (mBBr) as the capping agent (Fig. S9). We note that *V. cholerae* harbors complete GSH and coenzyme A (CoA) biosynthetic pathways from cysteine (Fig. S8*B*), thus the level of intracellular thiols is expected to be comparable to other γ-proteobacteria (82, 83). The basal organic thiol persulfide-to-thiol ratio are all below 0.5%, as is the inorganic disulfide/sulfide ratio, findings generally consistent with other γ-proteobacteria and other bacteria that possess other LMWTs as the most abundant thiol (22, 33, 73, 74) (Figs. 5 and S9*B*).

The addition of Na_2_S to *V. cholerae* cells leads to the expected transient increase on the GSSH levels (Figs. 5*D* and S10*A*) (22, 33), as well as a significant increase in the other organic and inorganic species detected in later time points (Figs. 5*C* and S10, *B* and *C*). The addition of Na_2_S results also in a significant increase in cellular cysteine and homocysteine levels possibly as a result of increased flux through cysteine synthase (CysK) (Figs. S9*B* and S10*A*), with a corresponding increase in cysteine persulfide as well as H_2_S (Figs. 5, *B* and *C* and S10*C*). Overall, our results suggest that the addition of exogenous sulfide results in its assimilation as organic thiol persulfides namely, GSSH, cysteine persulfide, as well as inorganic “sulfane” sulfur–bonded species, HSSH or S_2_. Strikingly, the addition of H_2_O_2_ results in an increase in intracellular sulfide and inorganic disulfide levels that nicely parallels that observed with exogenous Na_2_S treatment and is fully consistent with a previous study that showed that exogenous H_2_O_2_ treatment promotes endogenous H_2_S production (32) (Figs. 5, *B* and *C* and S10). Interestingly, we observe little to no corresponding increase in the organic LMWT or thiol persulfide levels upon H_2_O_2_ treatment, at least for cysteine or GSH (Figs. 5*D* and S10). This observation suggests that endogenous production of H_2_S does not necessarily have to impact the organic LMWT persulfide pool, a finding that suggests that HlyU is capable of sensing changes in either the inorganic or organic RSS pools. This result is consistent with prior findings with RSS sensors in other bacteria (36, 37); another possibility, not investigated here, is that HlyU reacts with protein persulfides or perhaps persulfidated H_2_S-producing enzymes themselves (66).

## Discussion

In this work, we show that the *V. cholerae* hemolysin activator HlyU possess the characteristics of a *bona fid*e persulfide sensor and reacts exclusively with “sulfane” sulfur donors to form a tetrasulfide bridge between its two cysteines, which abrogates DNA binding. While HlyU *in vitro* can distinguish between persulfides and other nonsulfur-containing oxidants, exogenous treatment of *V. cholerae* cells with either sulfide (Na_2_S) or H_2_O_2_ prevents HlyU-dependent expression of the *hlyA* operon. By means of quantitative RSS metabolite profiling experiments, we show that either sulfide (Na_2_S) or H_2_O_2_ increases the intracellular level of inorganic “sulfane” sulfur–bonded species, which we propose triggers HlyU dissociation from the promoter and subsequent recruitment of H-NS to reestablish silencing of *hlyA* expression. Based on our results and the current knowledge of *V. cholerae* colonization dynamics and exotoxin expression (1, 3, 32), we propose that RSS attenuation of HlyU-dependent *hlyA* expression may prevent undesired exotoxin release in the lumen of the gastrointestinal tract (a high H_2_S environment) (18, 84) and/or inflamed gut (high H_2_O_2_) (32). Thus, the intracellular RSS-specific regulation of HlyU may play a critical role in the proper spatiotemporal regulation of *hlyA* expression, allowing it exclusively when *V. cholerae* is at a preferred site of infection at the gut epithelia (3) (Fig. S11).

The remarkable functional diversity of the biochemically characterized ArsR family members in the RSS sensor cluster (Cluster 4, Fig. 1) illustrates the widespread importance of (per)sulfide signaling in bacteria. On the one hand, in free-living bacteria such as the purple bacterium *R. capsulatus*, persulfide sensing regulates sulfide-dependent photosynthesis (20, 76), while in plant symbionts and pathogens it is connected to biofilm formation and nodulation, shown in *Rhizobia* (56, 85). Human pathogens, such as *A. baumannii*, harness more than one persulfide-responsive regulator (the cluster 4 ArsR protein BigR and FisR, see Fig. 1*A*) and in this way may connect sulfide metabolism with biofilm formation and metal homeostasis (33). One of the cluster 4 members in *Escherichia coli* (YgaV) has been recently described as a master regulator connected to antibiotic resistance; however, its biochemistry remains unclear (86). In contrast, the enteric bacteria *Serratia marcescens* encodes PigS, a cluster 4 candidate persulfide sensor that has been shown to regulate the production of the red-pigmented antibiotic prodigiosin as well as sulfide metabolism genes (87). While PigS remains the sole characterized ArsR family protein with this function, antibiotic production regulation has also been shown for a known persulfide sensor from the CsoR family (88). These findings, that *bona fide* persulfide sensors regulate metabolic processes and pathways beyond sulfide detoxification, highlight the fact that “sulfane” sulfur species are not simply toxic molecules that bacteria need to metabolize and ultimately efflux, but may well also function as reporters critical for survival in a particular microenvironment.

To what extent sulfide signaling is restricted to the three described families of dithiol-based transcriptional regulators is still a matter of debate, as the experimental strategies to interrogate the crosstalk between persulfides and other redox signaling molecules are still being developed. It is not yet clear whether a prototypical heme–based or cysteine-based redox sensor capable of responding to extracellular sulfide is indeed part of the global response toward these species (39, 40, 41). Most of the experiments performed here and in previous work suggest that even *bona fide* persulfide sensors such as SqrR (36, 76) and CstR (37) react slowly with LMW persulfides. These moderate *in vitro* rates contrast with the rapid transcriptional response that these species trigger in cells, raising the possibility that persulfide-specific regulators respond to stable persulfidated protein thiols, instead of LMW persulfides (66). Addressing this question will provide new insights into the inducer specificity of thiol-based transcriptional regulators inside cells.

Moreover, while we have successfully correlated the increase in intracellular levels of “sulfane” sulfur–bonded species, these experiments make the prediction that HlyU would present in cells in the tetrasulfide upon sulfide or hydrogen peroxide treatment. Although recent advances in chemoproteomics have enabled intracellular detection of protein persulfidation (33, 34, 35, 67) even in a persulfide sensor from the CsoR family known to form various polysulfidated species *in vitro* (33, 35) these methods generally require a nucleophilic persulfidated or polysulfidated anion, and thus are less well suited to detect intramolecular polysulfide bridges unless they are in equilibrium with the “open” form. Future studies address the degree of persulfidation in *Vibrio* or other bacteria encoding *bona fide* persulfide sensors from the ArsR family will directly address this, while also exploring new experimental strategies to capture intramolecular polysulfides bridges (86).

Pathogen colonization and disease progression are determined by major biochemical changes within the host during enteric infection (17). Successful pathogens must sense and respond to these changes, which may be also accompanied by large perturbations in the microbiota, particularly induced by antibiotics (89). *Vibrio* spp. in the gut respond to these changes by inducing exotoxin expression (1, 32). Here, we show that intracellular persulfides formed in this sulfide-rich, oxidatively stressed inflamed gut (16, 17, 32) are capable of attenuating hemolysin expression. This may be beneficial for colonization and survival of the pathogen in the small intestine as it would prevent hemolysin expression in the lumen where the H_2_S concentration is high (∼1 mM) (18) but favor *hlyA* expression once the pathogen has reached the gut epithelium where the H_2_S concentration is significantly lower (Fig. S11). While it remains unclear what the most advantageous spatiotemporal *hlyA* expression pattern is, exotoxin expression in *V. cholerae* infectious cycle has been shown to occur predominantly in the last stage of colonization, where microcolony and proliferation occur at the gut epithelia (1, 3, 32). Thus, RSS inhibition of HlyU-dependent *hlyA* activation may be beneficial for survival, preventing expression of an exotoxin that can have a deleterious effect in the wrong stage of colonization. Future studies on the link between persulfide sensing and other adaptive responses in the infected host may help understand how other pathogens or pathobionts respond to biochemical signals in the gut or other infected tissues, where these RSS are prevalent and/or biosynthesized. Given the importance of (per)sulfide signaling, we expect that additional players in persulfide signaling and trafficking will continue to emerge as orchestrating distinct adaptative responses in bacteria.

## Experimental procedures

### *V. cholerae* strains and growth media

All *V. cholerae* strains used in this study are derivatives of strain E7946 (90), see Table S1. Mutant constructs were generated *via* splicing by overlap extension PCR and introduced into cells by chitin-dependent natural transformation exactly as previously described (91). *V. cholerae* strains used for qRT-PCR and metabolomic experiments were ΔCTX strains, lacking the gene coding for CT, so they could be cultured and harvested in a BLS2 laboratory. Routine culture employed LB medium at 30 °C for overnight growth and 37 °C with constant agitation. When appropriate, culture medium was supplemented with trimethoprim (10 μg/ml), carbenicillin (20 μg/ml), kanamycin (50 μg/ml), spectinomycin (200 μg/ml), and/or chloramphenicol (1 μg/ml).

### Protein preparation

*Vc*HlyU was subcloned into a pHis plasmid with NcoI and NedI encoding the untagged protein. *Vc*HlyU was expressed in *E. coli* BL21(DE3) cells at 16 °C overnight after induction with 1 mM IPTG. Freshly collected cells expressing *Vc*HlyU were suspended in 120 ml of buffer B (25 mM MES, 750 mM NaCl, 2 mM tris(2-carboxyethyl)phosphine (TCEP), 1 mM EDTA, pH 6) and lysed by sonication using a Thermo Fisher Scientific model 550 sonic dismembrator. TCEP was maintained in the buffers during the whole procedure to keep the only two cysteines (C38 and C104) in this protein reduced. The cellular lysate was centrifuged at 8000 r.p.m. for 15 min at 4 °C. The supernatant was collected and subjected to protein and nucleic acid precipitation by addition of 10% polyethylenimine (to 0.015% v/v) at pH 6. After stirring for 1 h at 4 °C, the solution was clarified by centrifugation at 8000 r.p.m. for 15 min at 4 °C, with the supernatant precipitated by the addition of (NH_4_)_2_SO_4_ to 70% saturation with stirring for 2 h. After centrifugation at 8000 rpm for 15 min, the precipitated protein was dissolved and dialyzed against buffer A (25 mM MES, 150 mM NaCl, 2 mM TCEP, 1 mM EDTA, pH 6). This solution was loaded onto a 10-ml sulfopropyl Fast Flow cation exchange column was equilibrated with buffer A. The protein was then eluted using a 150-ml linear gradient from 0.15 to 0.75 M NaCl. All the proteins characterized here were eluted as homodimers, as determined by a calibrated Superdex 75 (GE Healthcare) gel-filtration chromatography column (25 mM MES, 0.2 M NaCl, 2 mM EDTA, 2 mM TCEP, 5% glycerol, pH 6, 25 °C).

*Vc*HlyU samples for NMR experiments were isotopically labeled using isotopes for NMR experiments purchased from Cambridge Isotope Laboratories. Cells were grown in an M9 minimal media containing (per liter of growth media): 6 g Na_2_HPO_4_, 3 g KH_2_PO_4_, 0.5 g NaCl, 0.24 g MgSO_4_, 0.011 g CaCl_2_, 1 mg of thiamine, 2 g of ^13^C-glucose, 0.5 g ^15^N-NH_4_Cl, and 50 μg/ml ampicillin until an *A* of 0.6 was reached. Induction, expression, and purification conditions were the same as the above protocol.

### Fluorescence anisotropy–based DNA-binding experiments

Standard fluorescence anisotropy–based DNA-binding experiments were carried out using two 29-bp fluorescein (F)-labeled operator DNA fragments purchased at Integrated DNA Technologies, 5′F-(T) AT AAA TTA ATT CAG ACT AAA TTA GTT CAA A-3′ and its complement: 5′-TTT GAA CTA ATT TAG TCT GAA TTA ATT TAT A-3′ from the *hlyA* promoter region (hlyO), with a 10 nM concentration of DNA in DNA-binding buffer (10 mM Hepes, pH 7.0, 0.1 M NaCl, in the presence or in the absence of 2 mM TCEP). After the final addition of protein, *Vc*HlyU was treated with a 10-fold excess of “sulfane” sulfur in a GSSH-containing mixture or H_2_O_2_ with respect to the protein concentration (performed in the absence of reducing agent). Then, a 5 mM TCEP was added to determine if the oxidized protein could be reduced back to a DNA-competent oxidation state. All experiments were performed in triplicate. All anisotropy-based data were fitted to a simple 1:1, nondissociable dimer binding model to estimate *K*_a_ using DynaFit (www.biokin.com/dynafit) (92).

### LC-ESI-MS analysis of derivatized proteins

Reduced *Vc*HlyU was buffer exchanged anaerobically into a degassed 300 mM sodium phosphate buffer, pH 7.4, containing 1 mM EDTA. Reactions containing 60 μM protein were anaerobically incubated at room temperature with a 20-fold excess of oxidizing reagent, namely “sulfane” sulfur in a GSSH-containing mixture or H_2_O_2_ for 1 h or for different times as indicated in the figures. The reactions were quenched by addition of equal volumes of 60 mM IAM in the case of the RSSH or 60 mM of dimedone in the case of H_2_O_2_. Analysis was performed in the Laboratory for Biological Mass Spectrometry at Indiana University using a Waters Synapt G2S mass spectrometer coupled with a Waters ACQUITY Ultra Performance Liquid Chromatography I-Class system. Protein samples of 5 μl were loaded onto a selfpacked C4 reversed-phase column and chromatographed using an acetonitrile (ACN)-based gradient (solvent A: 0% ACN, 0.1% formic acid (FA); solvent B: 100% ACN, 0.1% FA). Data were collected and analyzed using MassLynx software (Waters) (https://www.waters.com/waters/en_US/MassLynx-Mass-Spectrometry-Software-/nav.htm?cid=513164&locale=en_US).

For aerobic conditions, *Vc*HlyU was exchanged into a 25 mM MES pH 6, 150 mM NaCl containing 1 mM EDTA. Reactions containing 60 μM protein were aerobically incubated at room temperature with a 20-fold excess of oxidizing reagent, namely “sulfane” sulfur or H_2_O_2_ for 1 h or overnight, as indicated in the figures. The reactions were quenched by addition of equal volumes of 60 mM IAM or dimedone. Protein preparations were sent to the Proteomics Core Facility of CEQUIBIEM at the University of Buenos Aires. Protein samples were purified and desalted using Stage tips C8 20 ul pipette tips (Thermo Fisher Scientific Cat # SP221). Stage tips were equilibrated with water containing 0.1% FA and samples were eluted in 10 ul of H_2_O:ACN:FA 40:60:0.1%. Samples were dried in speed vac and resuspended in water containing 0.1% FA. Proteins were analyzed with Orbitrap technology (Q-Exactive with High Collision Dissociation cell and Orbitrap analyzer), by direct injection and ionization was performed by electrospray ionization. Data was analyzed with the Xcalibur Software from Thermo Fisher Scientific (www.thermofisher.com/order/catalog/product/OPTON-30965).

### Preparation of RSSH-containing mixtures

GSSH, CysSSH and h-CysSSH were freshly prepared by mixing a 5-fold molar excess of freshly dissolved Na_2_S with the corresponding thiol disulfide, RSSR. and incubated anaerobically at 30 °C for 30 min in degassed 300 mM sodium phosphate (pH 7.4). The concentration of “sulfane” sulfur in the *in situ*–generated persulfides was determined using a cold cyanolysis assay as previously described (22), and these persulfide mixtures were used without further purification at the indicated final concentrations (76). While these mixtures contain inorganic polysulfides among the “sulfane” sulfur species, the molar fraction RSSH has been reported to be higher than 0.88 of “sulfane” sulfur species (76), which is also consistent with the speciation observed for our persulfidated isotopically labeled “heavy” standards (Fig. S12).

### P_h__l__y__A_-GFP fluorescence measurements

Strains were grown overnight in plain LB medium. Cells were then washed and concentrated in instant ocean medium (7 g/l; Aquarium Systems) to an *A*_600_ = 5. Then, 200 μl for each sample was transferred to a 96-well plate and GFP fluorescence was measured on a Biotek H1M plate reader. The background fluorescence was determined by using a WT E7946 strain (that lacks GFP) and subtracted from all samples.

### Quantitative real time PCR analysis

*V. cholerae* strains (Table S1) were inoculated from glycerol stocks into 5 ml LB medium and grown at 30 ºC overnight. The overnight culture was diluted 1/100 into 15 ml LB medium at a starting *A*_600_ ≈ 0.002, grown to an *A*_600_ of 0.2 at 37 °C, followed by the addition of stressor, Na_2_S (0.2 mM), or H_2_O_2_ (0.2 mM). An aliquot of the cultures was centrifuged for 10 min prior to the addition of stressor (*t* = 0), as well as 15 min and 60 min post addition of stressor to the cultures. Following centrifugation, the cell pellets were washed twice with ice-cold PBS, centrifuged for 5 min and stored at −80 °C until use. Pellets were thawed on ice and resuspended in 1 ml of TRI Reagent (catalog no. TR-118; Molecular Research Center). Resuspended cells were placed in tubes containing 0.1-mm silica beads (Lysing matrix B tubes, catalog no. 6911-100; MP Biomedicals) and lysed in a bead beater (Bead Ruptor 24 Elite; Omni) at a rate of 6 m/s for 45 s twice, with a 5-min cooling on ice between runs. Then, 200 μl of chloroform was added, followed by vigorous mixing and centrifugation for 15 min at 13,200 rpm. The top aqueous layer was removed to a new tube, and 70% ethanol was added at a 1:1 volume ratio. RNA purification was completed using the RNeasy minikit (catalog no. 74104; Qiagen) following DNase I treatment (catalog no. 79254; Qiagen). Next, 5 μg of total RNA was subsequently digested with the DNA-free kit (catalog no. AM1906; Ambion) and diluted 5-fold. First-strand complementary DNA (cDNA) was synthesized using random hexamers (Quanta Biosciences) and a qScript Flex cDNA synthesis kit (catalog no. 95049-100; Quanta Biosciences). Reactions contained 10 μl of 2× Brilliant III Ultra-Fast SYBR green QPCR master mix (catalog no. 600882; Agilent), 2 μl each of 2 μM PCR primers (see Table S1 for used primers), 0.3 μl of 2 μM ROX reference dye, and 6 μl of diluted cDNA. Relative transcript amounts were measured using the MX3000P thermocycler (Stratagene), running the SYBR Green with dissociation curve program and normalized to the amount of *recA*. The thermal profile contained 1 cycle at 95 °C for 3 min and 40 cycles at 95 °C for 20 s to 59 °C for 20 s. Subsequently, a dissociation curve starting at 55 °C going to 95 °C in 0.5 °C increments with a dwell time of 30 s was performed to assess the specificity of the reactions. Three biologically independent samples were measured for each treatment and the mean ± SD values are reported.

### Quantitation of cellular LMWTs and LMW persulfides

Overnight ΔCTX *V. cholerae* cells grown in LB media were diluted to an *A*_600_ of 0.02 in LB and grown in sealed tubes with constant agitation at 37 °C. When these cultures reached an *A*_600_ of 0.2, 0.2 mM Na_2_S or H_2_O_2_ was added. Samples were collected before (*t* = 0 min) addition of the stressor and at 15, 30, and 60 min, following addition of stressors. They were centrifuged at 3000 rpm for 10 min. The resulting pellets were washed with ice-cold PBS, pelleted again by centrifugation (16,100 rpm for 5 min), and stored frozen at −80 °C until use. Thawed cell pellets were resuspended in 200 μl of a PBS solution containing 3 mM of HPE-IAM labeling agent, or in 100 μl of mBBr solution containing 20 mM TRIS-HBr, pH 8, 50% ACN, and 1 mM mBBr. The resuspension solutions were then subjected to three freeze-thaw cycles in liquid nitrogen in the dark. Cell debris was removed by centrifugation, the supernatant was transferred to a 0.2 μm pore size centrifugal filter unit and centrifuged at 13,200 for 10 min prior to injection into a LC-MS system for quantitation of LMW thiol and persulfides as follows.

Samples (10 μl) were injected into a Triart C18 column (YMC, Inc) (50 by 2 mm inner diameter) and subjected to chromatography on a Waters Acquity Ultra Performance Liquid Chromatography I-class system, using a methanol-based gradient system (for solvent A, 10% methanol and 0.25% acetic acid, pH 3; for solvent B, 90% methanol and 0.25% acetic acid, pH 3) with the elution protocol at 25 °C and a flow rate of 0.2 ml/min as follows: at 0 to 3 min, 0% B isocratic; at 3 to 7 min, 0% to 25% B, linear gradient; at 7 to 9 min, 25% B isocratic; at 9 to 12 min, 25% to 75% B, linear gradient; at 12 to 14 min, 75% to 100% B, linear gradient; at 14 to 14.5 min, 100% B isocratic, followed by re-equilibration to 0% B. Quantitation of LMWTs and LMW persulfides was carried out with a Waters Synapt G2S mass spectrometer by spiking in a specific amount of LMW persulfide standards (HPE-IAM heavy) synthesized with deuterium isotopic labeling.

The persulfide standards used for quantification were GSSH, CysSSH, and h-CysSSH for their respective thiols and persulfides, and h-CysSSH for quantification of CoASH, CoASSH (mBBr labeling), and of inorganic sulfides and disulfides. These standards were obtained following the RSSH preparation described here and capped with HPE-IAM heavy, as follows. A 100 μM solution of each RSSH quantified by cold cyanolysis was mixed with 5 mM of HPE-IAM heavy and left for 1 h at 37 °C under anaerobic conditions. The degree of purity was evaluated by LC-MS (Fig. S12), as follows. A sample from each standard solution was spiked with 5 mM of HPE-IAM light and then injected these samples in the mass spectrometer. All the peaks present in each case were analyzed. The absence of the peak corresponding to the HPE-IAM-light–labeled metabolite accounts for completion of the reaction with HPE-IAM-heavy (Fig. S12 top panel), while the absence of other “sulfane” sulfur species is indicative of a good correspondence between the cold cyanolysis assay results and the concentration of persulfide (Fig. S12 bottom panels). Standards were used in all cases without further purification steps.

Analysis of peak areas was performed in Masslynx (v 4.1) software, and the data were normalized to protein concentrations measured using a Bradford assay with bovine serum albumin as the standard, as previously described (35). Data shown represent means and SDs of results from at least three biological replicates.

### SSN analysis

The EFI-EST webserver (https://efi.igb.illinois.edu/efi-est/) was used to generate an SSN using the Pfam PF01022 and Interpro IPR001845 databases of ArsR proteins as input, using the “families” option of the webserver (59). The initial computation parameters were left at their default values, with an E-value of 5. Given the large number of sequences used as input (365,837), an UniRef50 database was generated, where each node in the SSN groups sequences that share at least 50% of sequence identity. For the final calculation of the network, sequences shorter than 50 residues in length or longer than 150 residues were left out of the network, as such short sequences likely come from peptide fragments and those longer than 150 residues cannot correspond to the canonical ArsRs, which have only one domain (55). For the final network, an alignment score of 22 was used as threshold to cluster together UniRef50 nodes with at least 40% of sequence identity. The SSN was visualized using the Cytoscape software, version 3.8.0 (https://cytoscape.org/release_notes_3_8_0.html) (93). The network was represented using an unweighted prefuse force–directed layout. The network was colored, and the sequence logos and further cluster analysis were done using the “SSN utilities” of the EFI-EST webserver (59). A genome neighborhood analysis was performed in the EFI-GNT webserver (59) using subnetworks of the main network (Fig. S1*B*) form by each of the main (sub)clusters as input, with a neighborhood size of five and a minimal co-occurrence percentage lower limit of 5. Sequence logos for each cluster were obtained by performing a multiple sequence alignment (MSA) of the UNIREF50 sequences from each (sub)cluster using the MEGA software (version 11) (https://www.megasoftware.net/) (94) and the MUSCLE algorithm (95). The gap extension penalty was set to −0.5, and the neighbor-joining algorithm was selected as a cluster method in all iterations. All other parameters for the MSA were left in their default values. Alignments were manually curated by removing portions of the N terminal and C terminal in abnormally long sequences. Gaps accounting for indels in a minority of the sequences aligned were removed to avoid the overestimation of the conservation of residues in those positions in the sequence logos. Logos were generated using the Skylign web server (96) with default parameters using the MSAs exported from MEGA as inputs. The secondary structure prediction for the sequence logos shown in Figure 1*B* was obtained using the JPred4 webserver (97), using the consensus sequence for each cluster obtained in their MSA. All the consensus sequences from the studied clusters shared the same secondary structure prediction. Sequences within certain clusters in the network were assigned a putative function based on the information provided by their genome neighborhood, the conservation of key sequence motives within a cluster such as the DNA-binding residues (51) or the ligand-binding residues (55), and the regulatory function of characterized ArsRs that mapped within that cluster. Previously biochemically characterized ArsR family proteins (Table S2) 98, 99, 100, 101, 102, 103, 104, 105, 106, 107, 108, 109, 110, 111, 112, 113, 114, 115, 116, 117, 118, 119, 120, 121, 122, 123, 124, 125, 126 were found in the network by manually searching for their UniProt ID in Cytoscape.

### NMR spectroscopy

Typical NMR sample solution conditions were 200 μM ^15^N-labeled WT HlyU in 20 mM MES pH 6, 250 mM NaCl, 1 mM EDTA buffer as indicated. A Bruker 600 MHz spectrometer equipped with room temperature probe was used to acquire data for all HlyU samples. NMR data were processed using NMRPipe and were analyzed using Sparky. All spectra were acquired at 30 °C as indicated. Chemical shift is referenced relative to 2,2-dimethyl-2-silapentene-5-sulfonic acid.

### CD spectroscopy

The CD spectra were recorded at 25 °C using a JASCO-810 spectropolarimeter flushed with N_2_ and a 0.1 cm path length cuvette. Ten spectra were registered from 300 to 195 nm, at 0.1 nm intervals. Unfolding of secondary structure was followed by heating a protein solution (33 μM) from 25 to 90 °C and following the decrease in ellipticity at 235 nm using a 1 cm path length cuvette. A 25 mM Hepes, 200 mM NaCl, and 1 mM TCEP (reducing agent only added to the solution for the experiment in reduced HlyU) buffer was used in these experiments.

## Data availability

The authors declare that all data supporting the findings of this study are available within the paper and its Supplementary information files. All raw data are available from the corresponding author upon request.

## Supporting information

This article contains supporting information (33, 98, 99, 100, 101, 102, 103, 104, 105, 106, 107, 108, 109, 110, 111, 112, 113, 114, 115, 116, 117, 118, 119, 120, 121, 122, 123, 124, 125, 126).

## Conflict of interest

The authors declare that they have no conflicts of interest with the contents of this article.
