# Supplementary material for: Increased intracellular persulfide levels attenuate HlyU-mediated hemolysin transcriptional activation in Vibrio cholerae
Source: J Biol Chem. 2023 Aug 9;299(9):105147. doi: 10.1016/j.jbc.2023.105147 (PMC10509353; doi:10.1016/j.jbc.2023.105147)
Supplement: Supporting Figures S1–S12 [file mmc1.docx]

**Supporting Information**

**Increased intracellular persulfide levels attenuate HlyU-mediated hemolysin transcriptional activation in *Vibrio cholerae***

Cristian M. Pis Diez^1,2^, Giuliano T. Antelo^1,2^, Triana N. Dalia^3^, Ankur B. Dalia^3^, David P. Giedroc^2^* and Daiana A. Capdevila^1^*

^1^ Fundación Instituto Leloir, Instituto de Investigaciones Bioquímicas de Buenos Aires (IIBBA-CONICET), C1405BWE Ciudad Autónoma de, Buenos Aires, Argentina

^2^ Department of Chemistry, Indiana University, Bloomington, IN 47405-7102, USA

^3^ Department of Biology, Indiana University, Bloomington, IN 47405-7102, USA

**This file contains Supporting Figures S1-S10.**


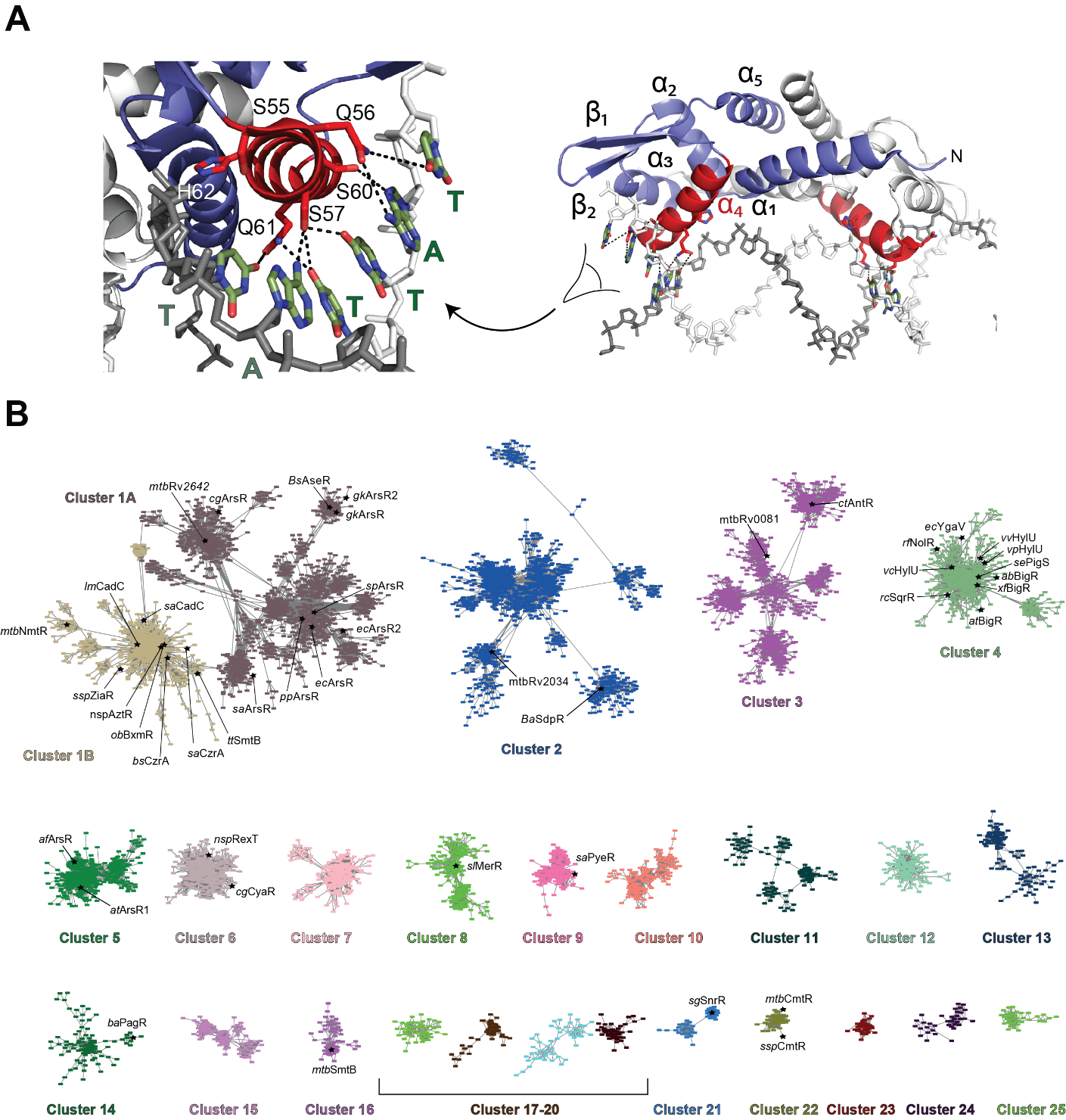


**Figure S1. (A)** ArsR superfamily proteins architecture (a1-a2-a3-a4-b1-b2-a5) and DNA recognition exemplified by the structure of DNA-bound NolR (4omy). The contacts between the protein sequence in α4 (*red*) and bases from DNA (*green*) in the first half-site are marked with *black* dashes. **(B)** All the main clusters generated in the sequence similarity network obtained using Pfam PF01022 and Interpro IPR001845 datasets of annotated ArsRs proteins.

**
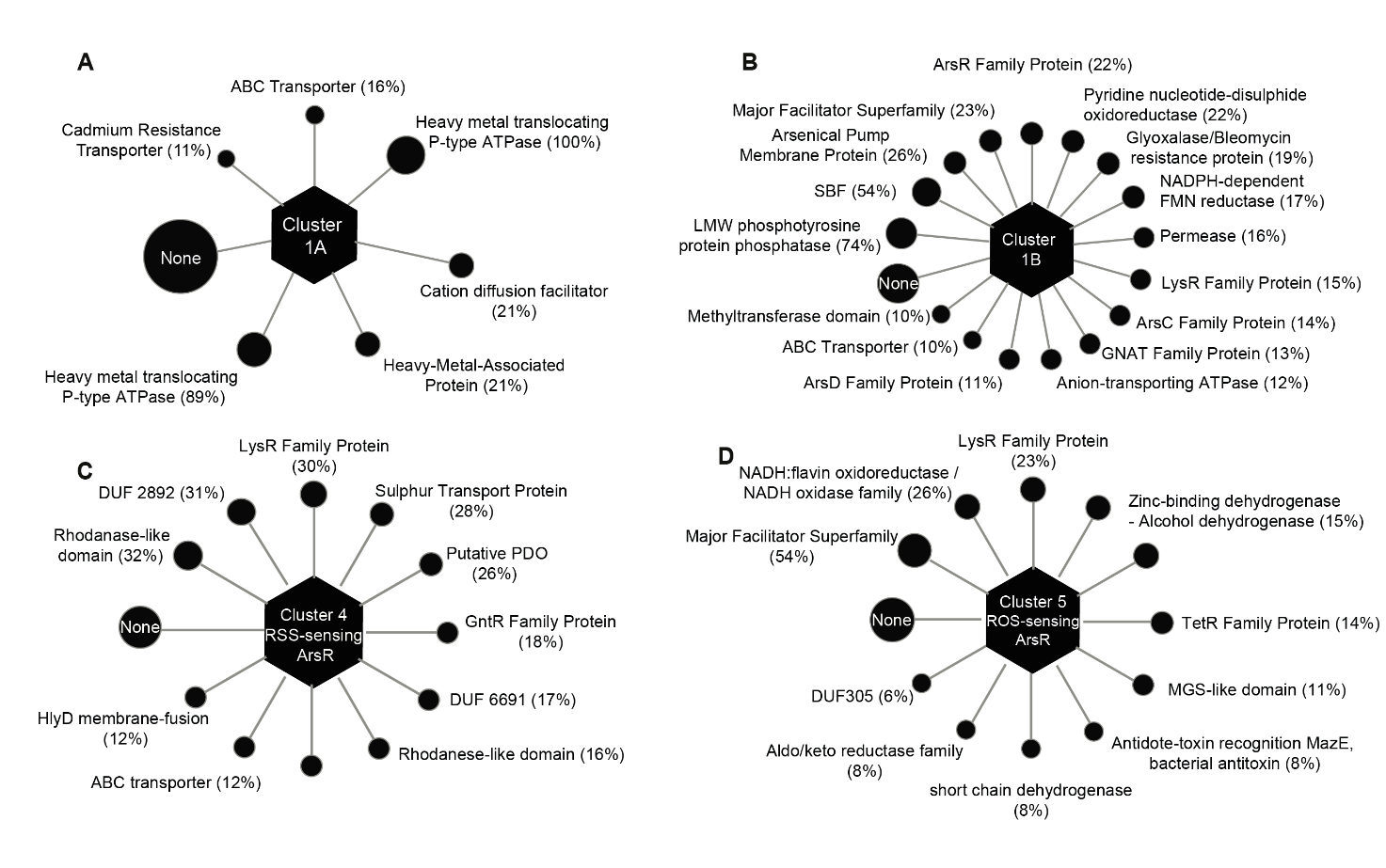
**

**Figure S2.** Genomic neighborhood analysis from cluster 1 (panels A and B), cluster 4 (panel C) and cluster 5 (panel D). The analysis involves the first five genes in the genomic neighborhood of each ArsR gene (upstream or downstream). Putative functions of encoded proteins were inferred from the Pfam annotated domain functions. The large filled circles emanating from each cluster represent the most frequent co-occurrence genes with a given function, and in parentheses, the percentage of co-occurrence is given. For example, in cluster 1A, 89% of the ArsR have a neighbor protein containing a domain associated with heavy-metal translocating ATPase. We note that the neighborhood of a given ArsR in a cluster might contain multiple genes with the same associated function.

**
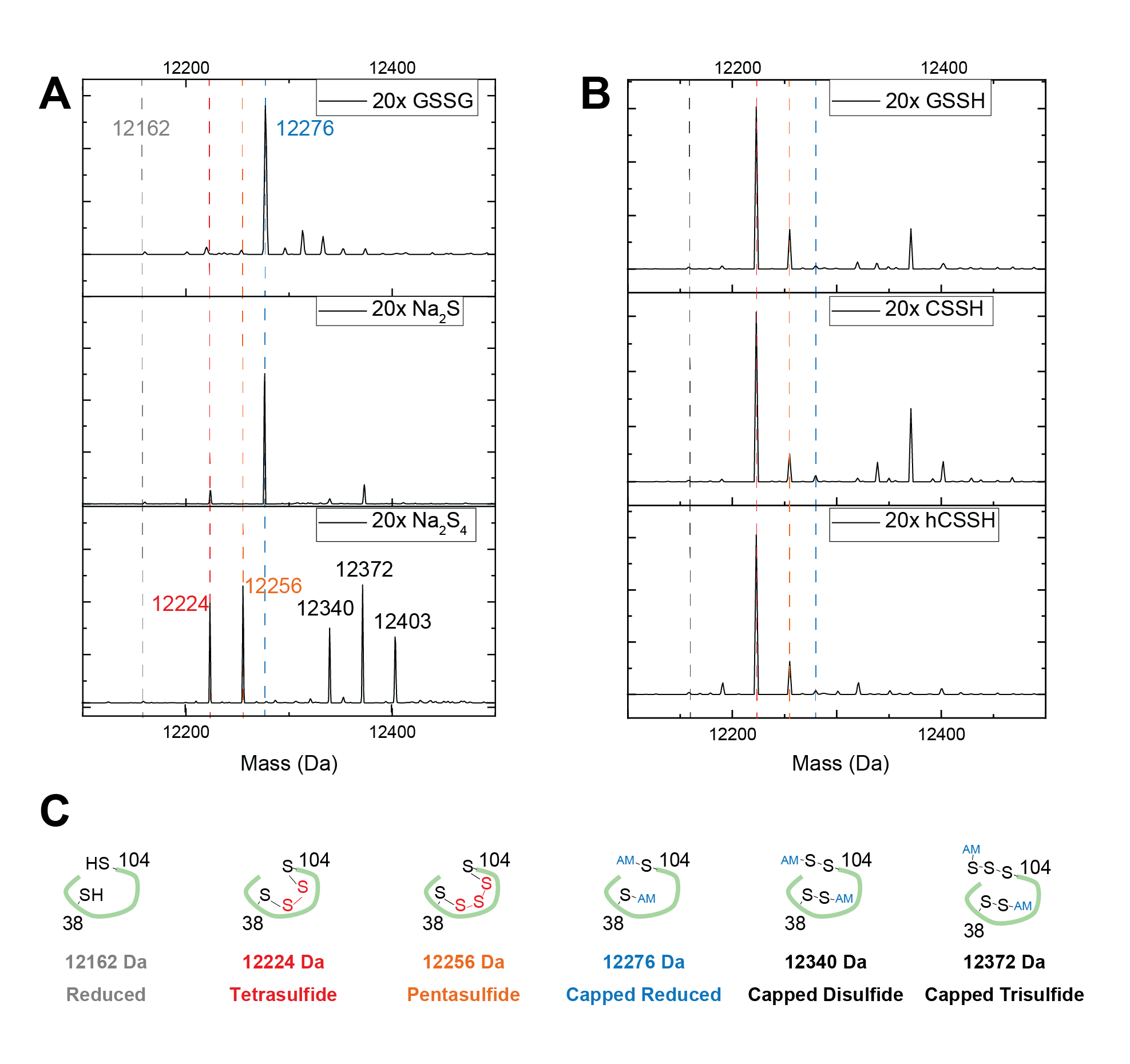
**

**Figure S3.** LC-ESI-MS analysis of HlyU *in vitro* reactivity upon a one-hour incubation with a 20-fold excess of **(A)** GSSG, Na_2_S and Na_2_S_4_ and **(B)** organic persulfides GSSH, cysteine and homocysteine persulfides (CSSH and hCSSH, respectively) and then capped with IAM. *Grey* dashed lines correspond to the reduced and uncapped HlyU monomer, while the two *red* dashed lines correspond to the tetrasulfide and pentasulfide species. (**C**) Schematic representation of the different states of HlyU captured in this experiment and their masses.

**
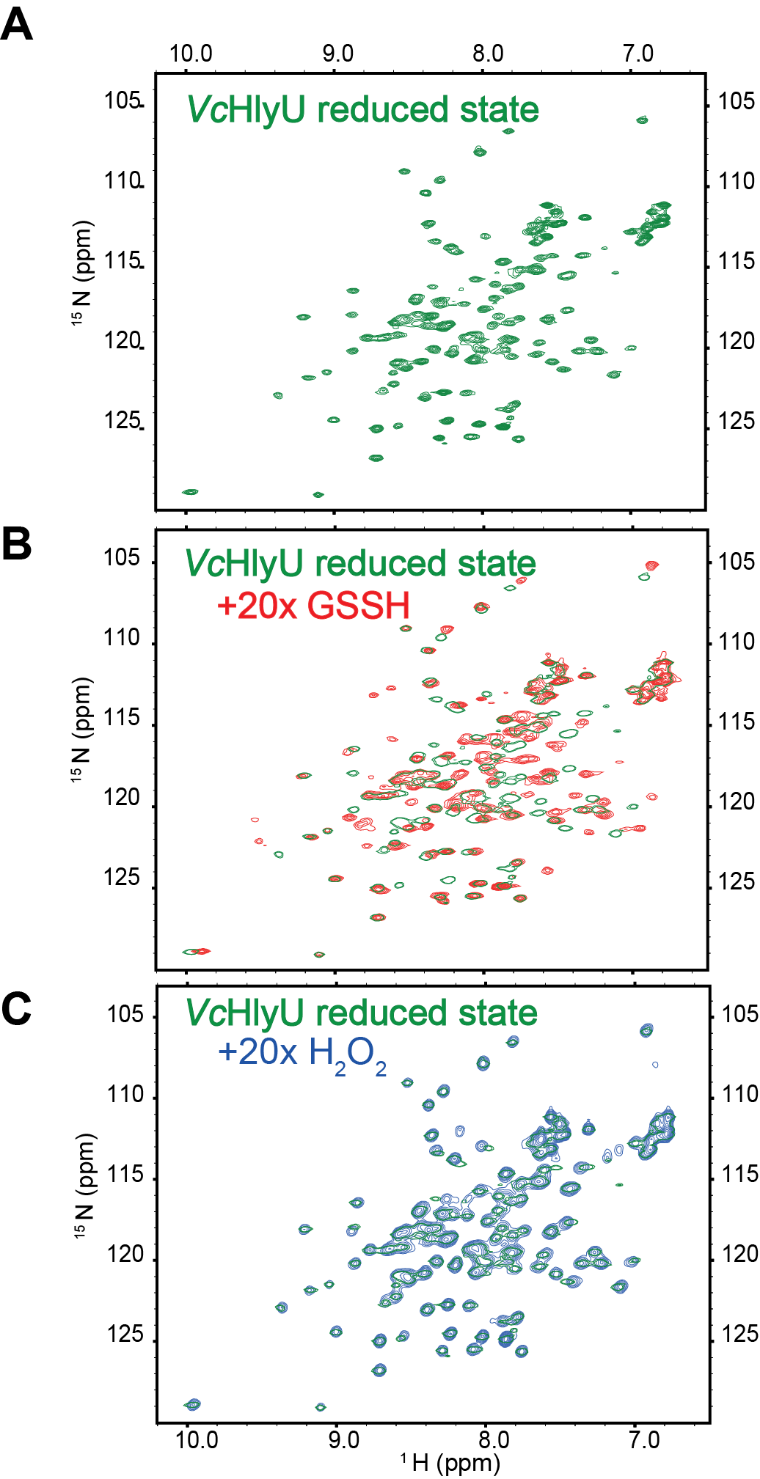
**

**Figure S4. (A)** ^1^H, ^15^N HSQC spectrum of reduced *Vibrio cholerae* HlyU. **(B)** Overlay of ^1^H, ^15^N HSQC spectrum of reduced *Vibrio cholerae* HlyU (*green*) and pre-treated with 20x GSSH (*red*). **(C)** Overlay of ^1^H, ^15^N HSQC spectrum of reduced *Vibrio cholerae* HlyU (*green*) and pre-treated with 20x H_2_O_2_ (*blue*). All spectra were measured at 30 ºC using 200 μM protein in 20 mM MES pH 6, 250 mM NaCl, 1 mM EDTA buffer, with the addition of 2 mM TCEP in the case of the reduced state.

**
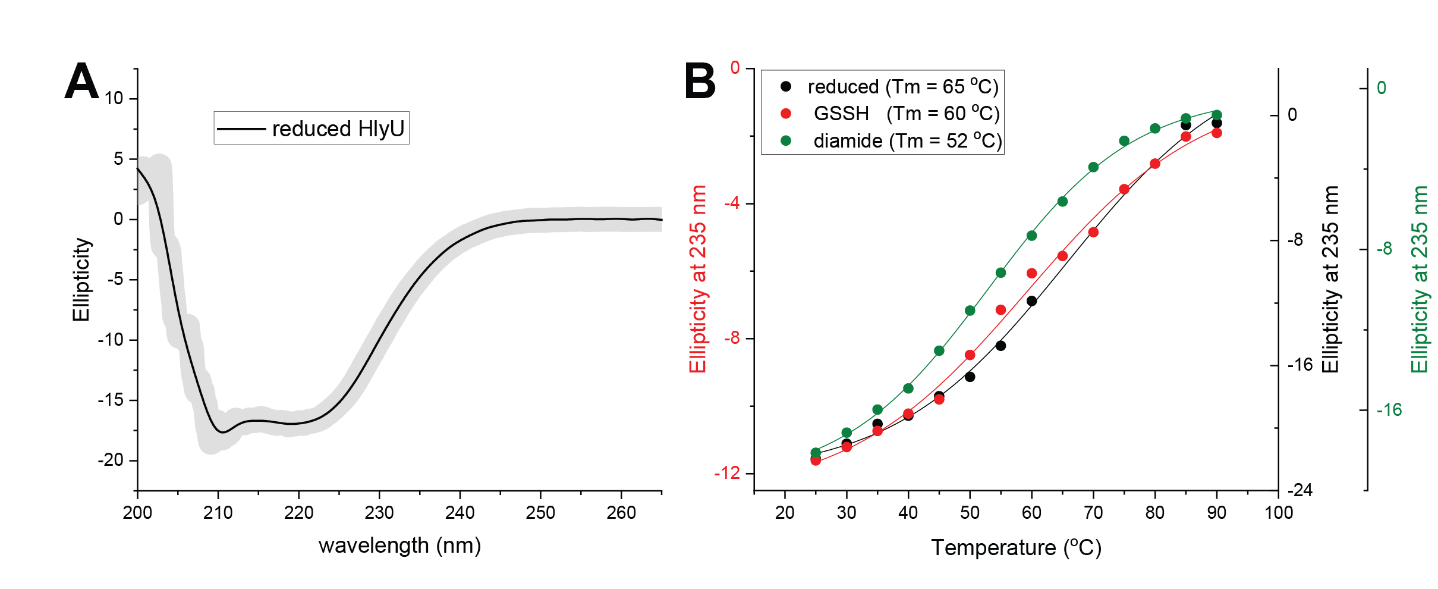
**

**Figure S5. (A)** Far-UV CD spectra of reduced HlyU. **(B)** Temperature-induced conformational transitions observed as changes in ellipticity at 232 nm in the 25–90 ºC temperature range for reduced (*black*), tetrasulfide (*red*) and diamide treated (disulfide, *green*) crosslinked HlyU. The line indicates a sigmoidal fitting used to obtain the melting temperature. All spectra were measured using 33 μM protein in 25 mM HEPES, pH 7.0, 200 mM NaCl, 1 mM EDTA, with the addition of 1 mM TCEP in the case of the reduced state.

**
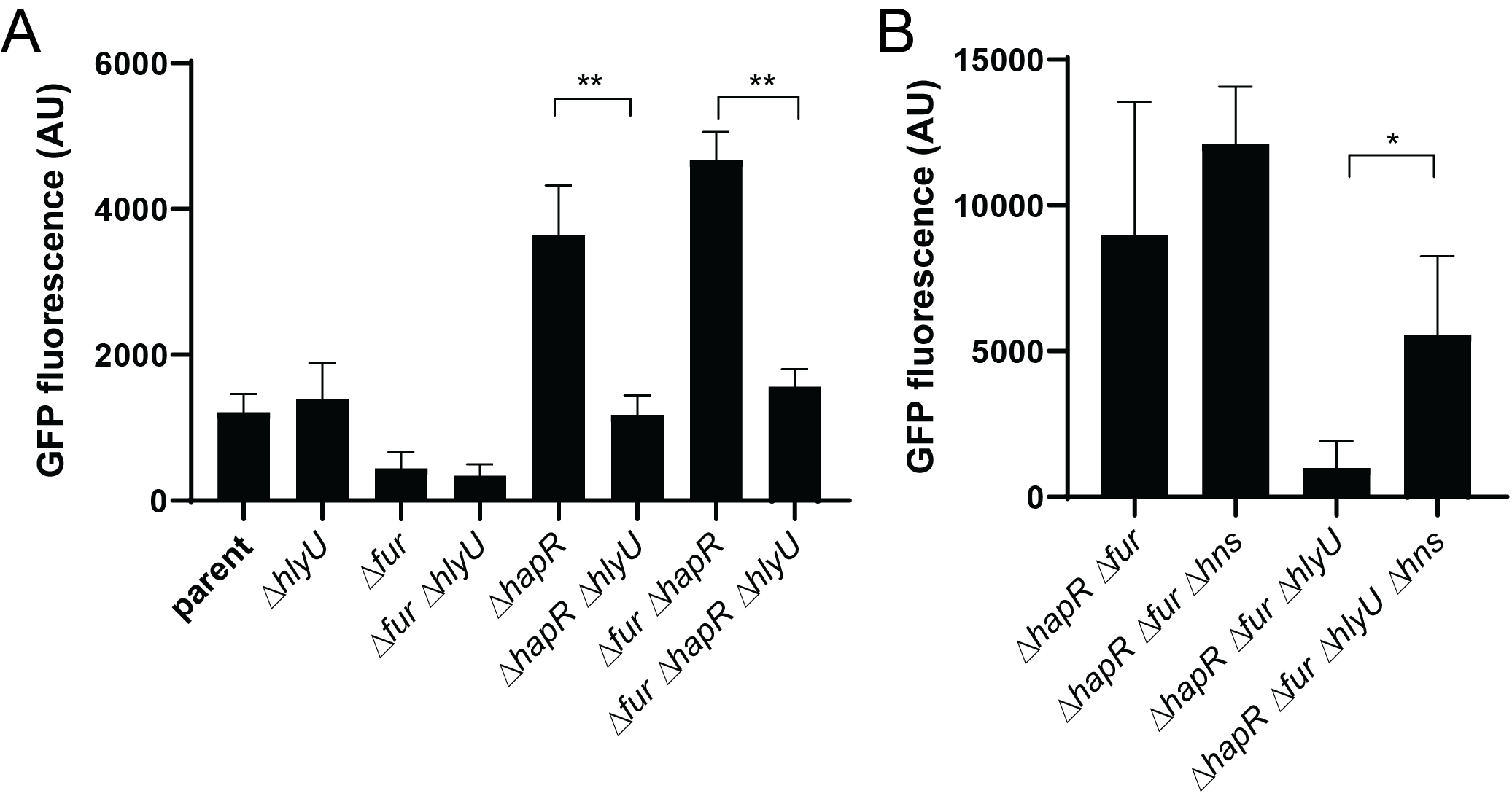
Figure S6.** Assessing HlyU activity *in vivo* using a P_hlyA_-GFP transcriptional reporter. Fluorescence of the indicated *V. cholerae* strains was measured to assess the impact of **(A)** HapR / Fur, and **(B)** HNS on HlyU-dependent activation of P_hlyA_. Data are from four independent biological replicates and shown as the mean ± SD. Statistical significance was established using an unpaired parametric *t*-test (**p<0.01, *p<0.05).

**
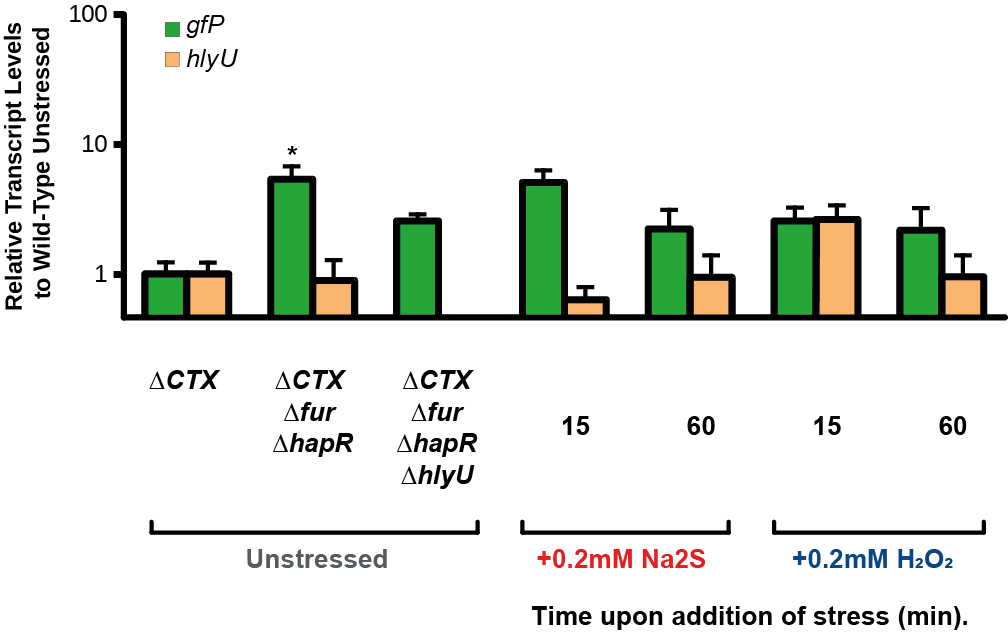
**

**Figure S7.** Fold changes transcripts levels of *Vc* *hlyU* and *gfP* followed by quantitative RT-PCR performed over a ΔCTX Δfur ΔhapR *V. cholerae* strain with the addition of Na_2_S or H_2_O_2_. Transcript values were normalized relative to the transcription level of *recA*. The values correspond to transcript levels relative to wild-type unstressed (WT UN) and are shown as mean ± SD from replicate cultures. Statistical significance was stablished using a paired t test relative to WT UN under the same conditions (**p<0.01, *p<0.05).

**
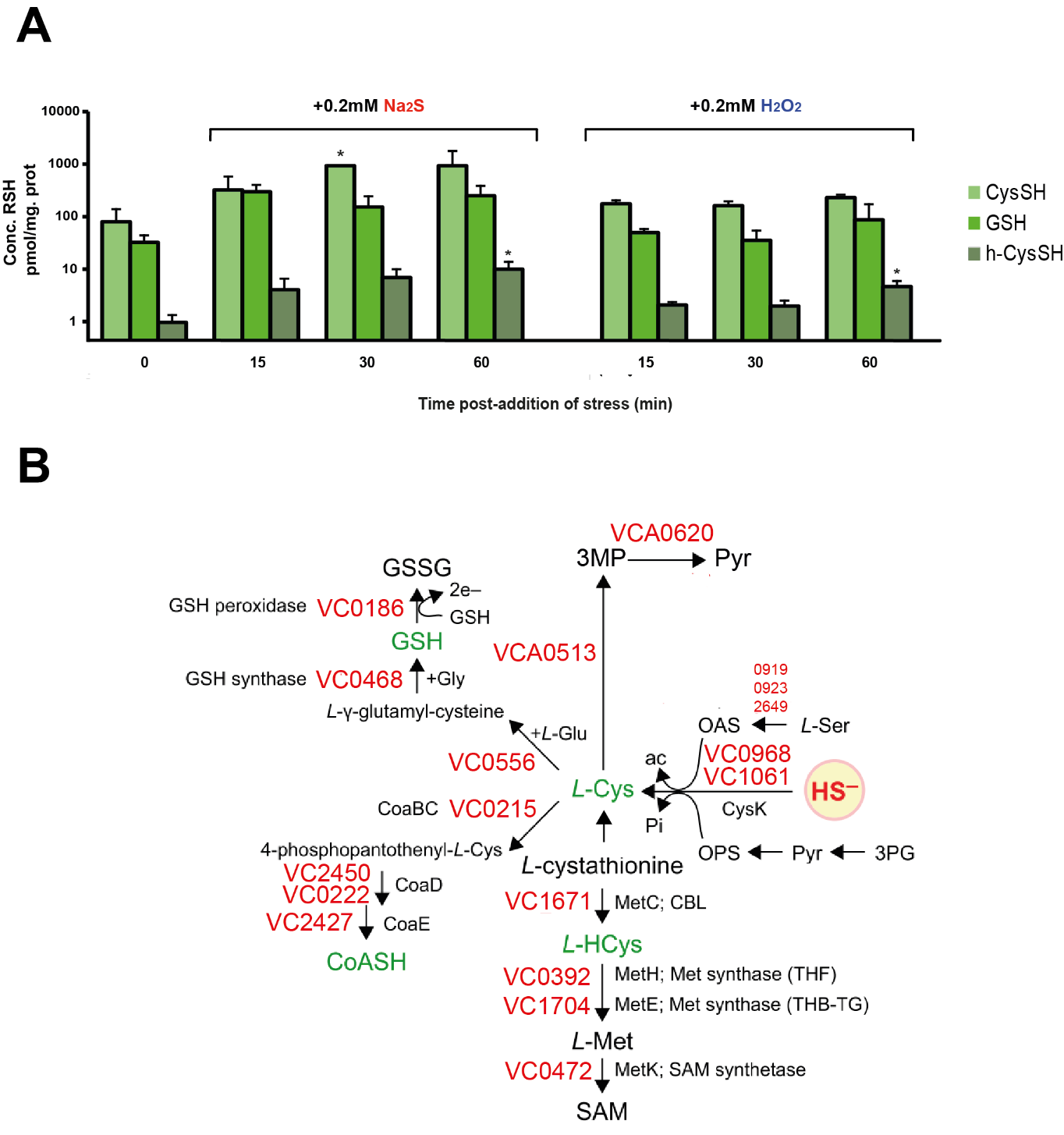
**

**Figure S8. (A)** Endogenous concentrations of LMW thiols before and after the addition of Na_2_S and H_2_O_2_ to mid-log-phase cultures (*, P< 0.05 using a paired t test relative to WT UN under the same conditions) determined using HPEIAM as capping agent. **(B)** *V. cholerae* genes encoding proteins associated with the biosynthesis of LMW thiols. OPS, O-phospho-L-serine; OAS, O-acetyl-L-serine; ac, acetate; pyr, pyruvate; CBL, cystathonine-γ-lyase. Adapted from reference (33).

**
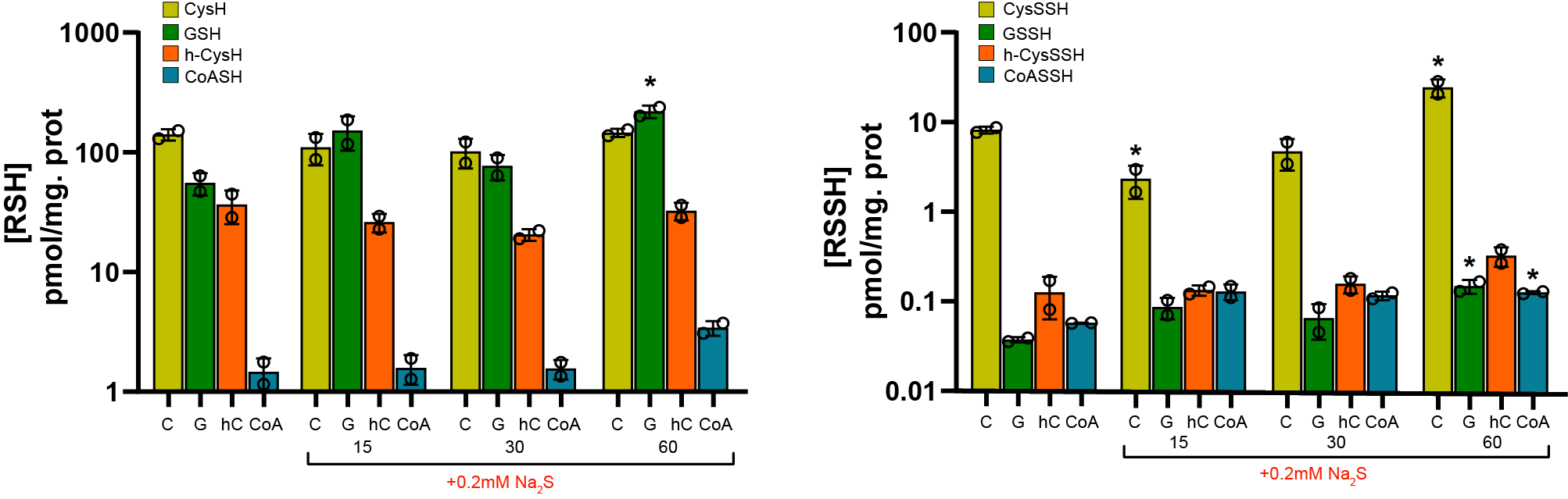
**

**Figure S9. (A)** Endogenous concentrations of LMW thiols before and after the addition of 0.2 mM Na_2_S to mid-log-phase cultures (*, p< 0.05) determined using mBBr as capping agent. **(B)** Endogenous concentrations of LMW persulfides before and after the addition of Na_2_S to mid-log-phase cultures (*, p< 0.05 using a paired t test relative to WT UN under the same conditions) determined using mBBr as capping agent.


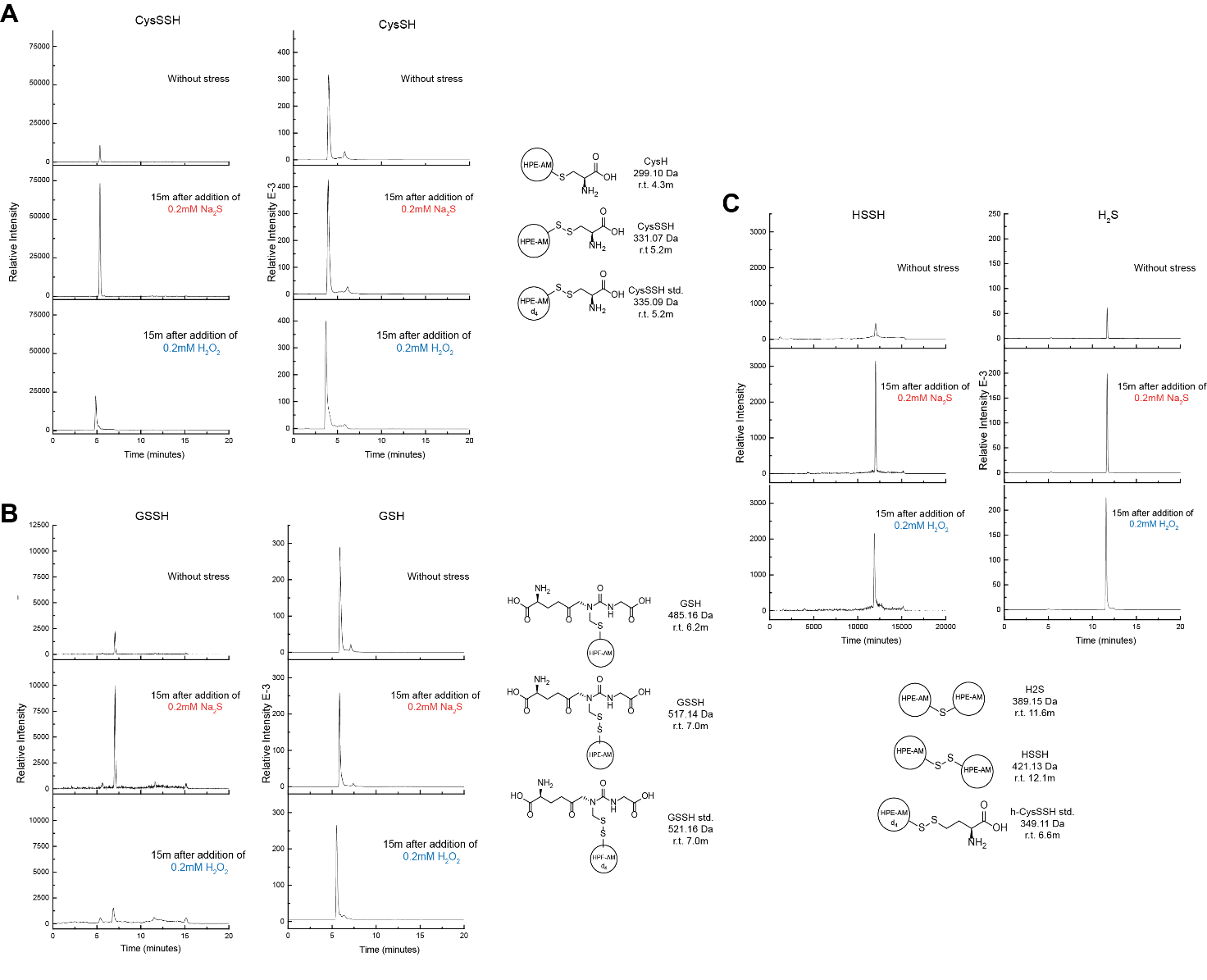


**Figure S10. (A)** Extracted ion chromatograms corresponding to cysteine thiol and cysteine persulfide (top panel) in unstressed cells and following 15 min of applied stress (Na_2_S, middle panel and H_2_O_2_, bottom panel). **(B)** Extracted ion chromatograms corresponding to glutathione thiol and glutathione persulfide. **(C)** Extracted ion chromatograms corresponding to inorganic sulfide and disulfide. The peaks show in each case the relative intensity of the HPE-IAM labelled metabolites, relative to the intensity of the internal standard. On the right, we show the molecular structure, molecular weight and retention time of the metabolites analyzed in the panels, together with the internal standard used in each case.

**
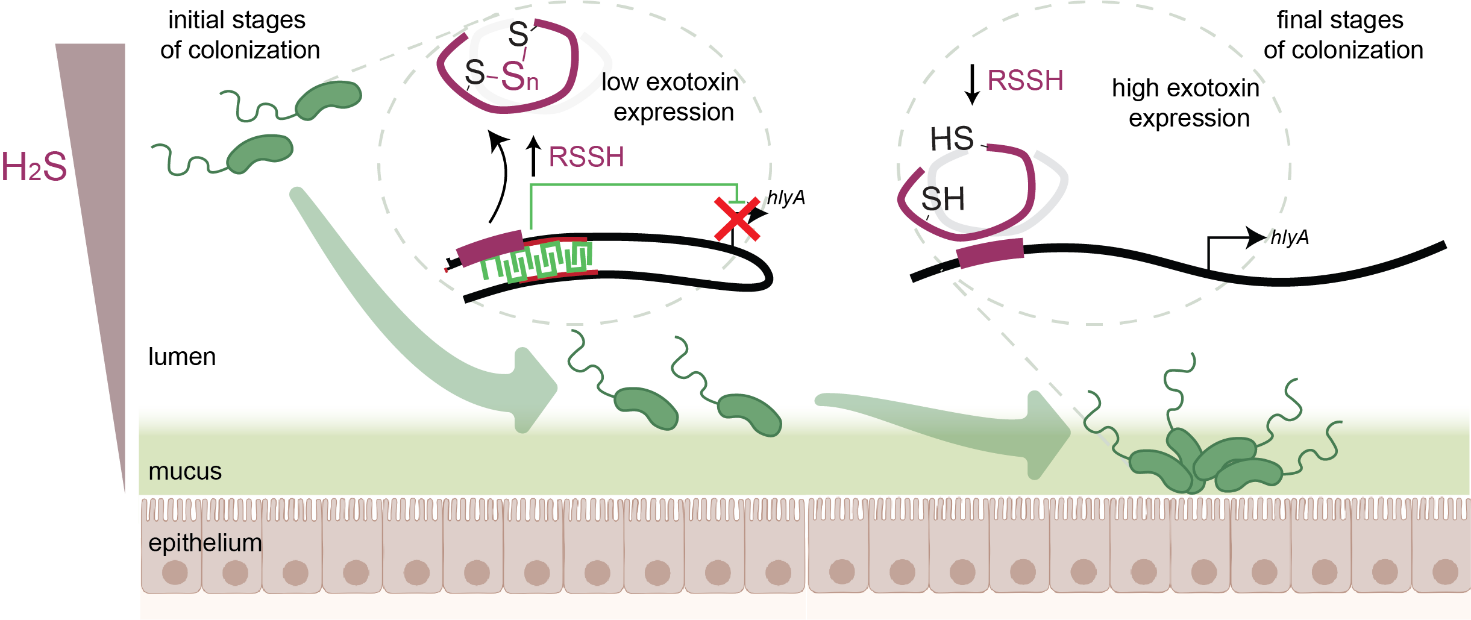
Figure S11.** A proposed mechanism for the beneficial effects of RSS dependent HlyU-mediated attenuation of *hlyA* expression when *V. cholerae* is exposed to high H2S concentration in the lumen of the gastrointestinal tract. HlyU-mediated HNS eviction occurs when *V. cholerae* reaches the gut epithelium.

**
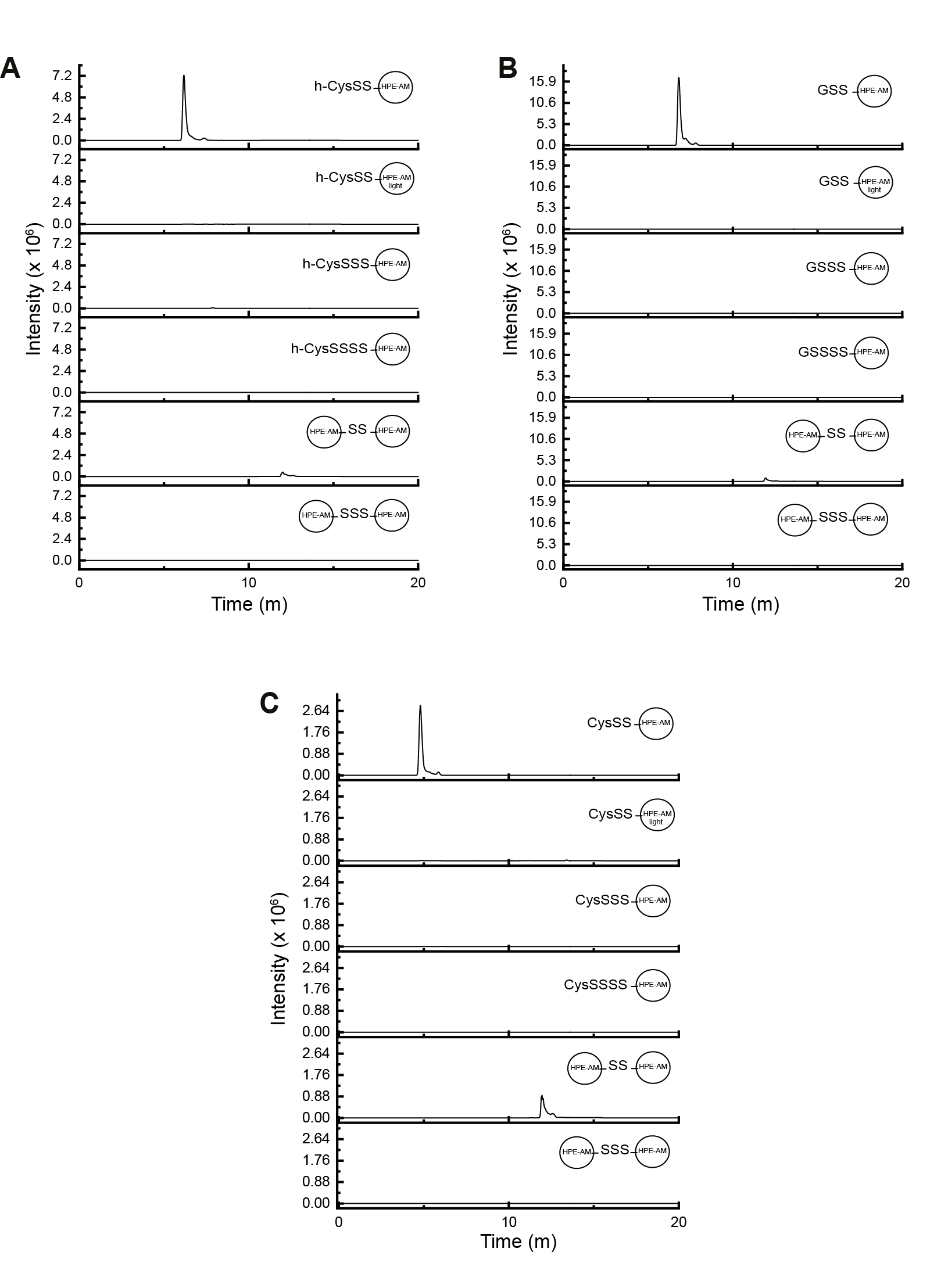
**

**Figure S12.** Extracted ion chromatograms from the standard solutions of (**A**) hCysSSH, (**B**) GSSH, (**C**) CysSSH reacted with isotopically “heavy” (*d*_4_) HPE-IAM (see Materials and Methods). Each panel shows, from top to bottom, the extraction of the mass feature corresponding to RSS-HPEAM, RSS-HPEAM (light), RSSS-HPEAM, RSSSS-HPEAM, and inorganic disulfides and trisulfides. As can be seen, more than 90% of the sulfane sulfur quantified by cold cyanolysis is accounted for by the corresponding organic persulfide that we used as a standard in this work.
