# Supplementary material for: Increased intracellular persulfide levels attenuate HlyU-mediated hemolysin transcriptional activation in Vibrio cholerae
Source: J Biol Chem. 2023 Aug 9;299(9):105147. doi: 10.1016/j.jbc.2023.105147 (PMC10509353; doi:10.1016/j.jbc.2023.105147)
Supplement: Supporting Table S1 [file mmc2.docx]

**Supporting Information**

**Increased intracellular persulfide levels attenuate HlyU-mediated hemolysin transcriptional activation in *Vibrio cholerae***

Cristian M. Pis Diez^1,2^, Giuliano T. Antelo^1,2^, Triana N. Dalia^3^, Ankur B. Dalia^3^, David P. Giedroc^2^* and Daiana A. Capdevila^1^*

^1^ Fundación Instituto Leloir, Instituto de Investigaciones Bioquímicas de Buenos Aires (IIBBA-CONICET), C1405BWE Ciudad Autónoma de, Buenos Aires, Argentina

^2^ Department of Chemistry, Indiana University, Bloomington, IN 47405-7102, USA

^3^ Department of Biology, Indiana University, Bloomington, IN 47405-7102, USA

**This file contains Supporting Tables S1.**

**Table S1:** *V. cholerae* strains and primers used for qRT PCR used for this study.

| **Strain** | **Description** |
| --- | --- |
| TND0004 | *WT* |
| TND2438 | *∆lacZ::P_hlyA_-msfGFP Cm^R^* |
| TND2455 | *ΔhlyU::Kan^R^, ∆lacZ::P_hlyA_-msfGFP Cm^R^* |
| TND2459 | *ΔhapR::Spec^R^, ∆lacZ::P_hlyA_-msfGFP Cm^R^* |
| TND2456 | *ΔhlyU::Kan^R^, ΔhapR::Spec^R^, ∆lacZ::P_hlyA_-msfGFP Cm^R^* |
| TND2533 | *Δfur::Tm^R^, ΔhapR:Spec^R^, ∆lacZ::P_hlyA_-msfGFP Cm^R^* |
| TND2534 | *Δfur::Tm^R^, ΔhapR::Spec^R^, ΔhlyU::Kan^R^, ∆lacZ::P_hlyA_-msfGFP Cm^R^* |
| TND2531 | *Δfur:Tm^R^, ∆lacZ::P_hlyA_-msfGFP Cm^R^* |
| TND2532 | *Δfur:Tm^R^, ΔhlyU:Kan^R^, ∆lacZ::P_hlyA_-msfGFP Cm^R^* |
| TND3182 | *ΔhapR:Spec^R^, Δfur::Tm^R^, ∆lacZ::P_hlyA_-msfGFP Cm^R^* |
| TND3183 | *ΔhapR:Spec^R^, Δfur::Tm^R^, ∆hns::Carb^R^, ∆lacZ::P_hlyA_-msfGFP Cm^R^* |
| TND3185 | *ΔhapR:Spec^R^, Δfur::Tm^R^, ∆hlyU::Kan^R^, ∆lacZ::P_hlyA_-msfGFP Cm^R^* |
| TND3186 | *ΔhapR:Spec^R^, Δfur::Tm^R^, ∆hlyU::Kan^R^, ∆hns::Carb^R^, ∆lacZ::P_hlyA_-msfGFP Cm^R^* |
| **Primers PCR** | **Sequence** |
| *recA* – rev | GCGCAGCAATCTTGTTCTTC |
| *recA* – fwd | CGTTTGGATATTCGCCGTACT |
| *hlyA* – rev | CTC TGT GGC TGA GGC TTT AT |
| *hlyA* – fwd | CGA TGC TTT GTG GGT GAA TAC |
| *GFP* – rev | GCTCTTGCACGTATCCTTCT |
| *GFP* – fwd | TTGTGACGACTCTGACTTATGG |
| *hlyU* – rev | CCA CGC TAG ATG TTG AGA AAG A |
| *hlyU* – fwd | GGA CAA TGA ACT GTC GGT AGG |
