# Supplementary material for: Increased intracellular persulfide levels attenuate HlyU-mediated hemolysin transcriptional activation in Vibrio cholerae
Source: J Biol Chem. 2023 Aug 9;299(9):105147. doi: 10.1016/j.jbc.2023.105147 (PMC10509353; doi:10.1016/j.jbc.2023.105147)
Supplement: Supporting Table S2 [file mmc3.docx]

**Supporting Information**

**Increased intracellular persulfide levels attenuate HlyU-mediated hemolysin transcriptional activation in *Vibrio cholerae***

Cristian M. Pis Diez^1,2^, Giuliano T. Antelo^1,2^, Triana N. Dalia^3^, Ankur B. Dalia^3^, David P. Giedroc^2^* and Daiana A. Capdevila^1^*

^1^ Fundación Instituto Leloir, Instituto de Investigaciones Bioquímicas de Buenos Aires (IIBBA-CONICET), C1405BWE Ciudad Autónoma de, Buenos Aires, Argentina

^2^ Department of Chemistry, Indiana University, Bloomington, IN 47405-7102, USA

^3^ Department of Biology, Indiana University, Bloomington, IN 47405-7102, USA

**This file contains Supporting Tables S2.**

**Table S2:** Characterized ArsRs in the SSN.

| **Protein Name*** | **Organism** | **Uniprot ID** | **Cluster number in Network** |
| --- | --- | --- | --- |
| ecArsR (99) | *Escherichia coli* | P37309 | 1A |
| ArsR2 (100) | *Escherichia coli* | A0A142BMN6 | 1A |
| ArsR(101) | *Staphylococcus aureus* | P30338 | 1A |
| ArsR1&2 (102) | *Geobacillus kaustophilus* | Q5KUX7 | 1A |
| ArsR (103) | *Pseudomonas putida* | Q88LK1 | 1A |
| AseR(52) | *Bacilus subtilis* | P96677 | 1A |
| ArsR (62) | *Corynebacterium glutamicum* | [A0A5H1ZR36](https://www.rcsb.org/uniprot/A0A5H1ZR36) | 1A |
| Rv2642(104) | *Mycobacterium tuberculosis* | P71941 | 1A |
| AztR(105) | *Cyanobacterium anabaena* | Q8ZS91 | 1B |
| BxmR(106) | Oscillatoria brevis | Q76L30 | 1B |
| NmtR(107) | *Mycobacterium tuberculosis* | O69711 | 1B |
| ZiaR(108) | *Synechocystis sp.* | Q55940 | 1B |
| CadC(109) | *Staphylococcus aureus* | [P20047](https://www.rcsb.org/uniprot/P20047) | 1B |
| SmtB(110) | *Synechococcus elongatus* | [P30340](https://www.rcsb.org/uniprot/P30340) | 1B |
| CzrA(111) | *Staphyloccocus aureus* | [O85142](https://www.rcsb.org/uniprot/O85142) | 1B |
| CzrA(52) | *Bacilus subtilis* | O31844 | 1B |
| CadC(112) | *Listeria innocua serovar 6* | [P0A4U2](https://www.uniprot.org/uniprot/P0A4U2) | 1B |
| SmtB(113) | *Thermus thermophilus* | Q72KG0 | 1B |
| CadC (112) | *Lysteria monocytogenes* | [Q56405](https://www.uniprot.org/uniprot/Q56405) | 1B |
| Rv2034(57) | *Mycobacterium tuberculosis* | O53478 | 2 |
| SdpR (114) | *Bacilus subtilis* | O32242 | 2 |
| Rv0081(63) | *Mycobacterium tuberculosis* | P9WMI7 | 3 |
| AntR(115) | *Comamonas testosteroni* | A0A096FLR2 | 3 |
| BigR(33) | *Acinetobacter baumanni* | D0C7U0 | 4 |
| YgaV(116) | *Escherichia coli* | P77295 | 4 |
| NolR(56) | *Rhizobium fredii* | Q83TD2 | 4 |
| SqrR(36) | *Rhodobacter capsulatus* | [D5AT91](https://www.rcsb.org/uniprot/D5AT91) | 4 |
| HlyU(7)** | *Vibrio cholerae serotype O1* | P52695 | 4 |
| HlyU(13) | *Vibrio parahaemolyticus* | [Q87S95](https://www.uniprot.org/uniprot/Q87S95) | 4 |
| HlyU(12) | *Vibrio vulnificus* | A0A3Q0L222 | 4 |
| BigR(24) | *Xylella fastidiosa* | Q9PFB1 | 4 |
| BigR(117) | *Agrobacterium tumefaciens* | [Q8UAA8](https://www.uniprot.org/uniprot/Q8UAA8) | 4 |
| PigS (87) | *Serratia* sp. strain ATCC 39006 | E7BBJ0 | 4 |
| SoxR(118) | *Pseudaminobacter salicylatoxidans* | [Q5ZQN5](https://www.uniprot.org/uniprot/Q5ZQN5) | 4 |
| ArsR(62) | *Acidithiobacillus ferrooxidans* | [B7J952](https://www.rcsb.org/uniprot/B7J952) | 5 |
| ArsR(119) | *Agrobacterium tumefaciens ArsR 1* | H0HHH0 | 5 |
| CyeR(120) | *Corynebacterium glutamicum* | A4QI86 | 6 |
| YczG(121) | *Bacilus subtilis* | O31480 | 6 |
| RexT(46) | Nostoc sp. | Q8YVV6 | 6 |
| MerR(122) | *Streptomyces lividans* | P30346 | 8 |
| PyeR(123) | *Pseudomonas aeruginosa* | Q9HW47 | 9 |
| KmtR(124) | *Mycobacterium tuberculosis* | O53838 | 10 |
| PagR(125) | *Bacillus anthracis* | O31178 | 14 |
| SmtB(126) | *Mycobacterium tuberculosis* | P9WMI4 | 16 |
| SrnR(58) | *Streptomyces griseus* | Q8L1Y3 | 21 |
| CmtR(60) | *Streptomyces coelicolor* | [Q9RD34](https://www.uniprot.org/uniprot/Q9RD34) | 22 |
| CmtR(127) | *Mycobacterium tuberculosis* | P9WMI8 | 22 |

* The protein name is followed by the most updated publication with the available biochemistry information. ** This work.
